# Taurine Attenuates the Hypotaurine-Induced Progression of CRC *via* ERK/RSK Signaling

**DOI:** 10.3389/fcell.2021.631163

**Published:** 2021-04-15

**Authors:** Xiaodan Hou, Junwei Hu, Xinyu Zhao, Qing Wei, Rongping Zhao, Min Li, Qiong Li

**Affiliations:** ^1^Department of Laboratory Medicine, Renji Hospital, School of Medicine, Shanghai Jiaotong University, Shanghai, China; ^2^Suzhou Institute of Systems Medicine, Center of Systems Medicine, Chinese Academy of Medical Sciences, Suzhou, China; ^3^Department of Gastroenterology, Shanghai University of Medicine and Health Sciences Affiliated Zhoupu Hospital, Shanghai, China

**Keywords:** taurine, hypotaurine, tumor progression, ERK, colorectal cancer, prognosis

## Abstract

Colorectal cancer (CRC) is one of the most common malignant tumors, and previous metabolomics work has demonstrated great promise in identifying specific small molecules of tumor phenotype. In the present study, we analyzed the metabolites of resected tissues through gas chromatography-mass spectrometry (GC-MS), and found that the concentration of taurine in CRC tissues diminished whereas the concentration of hypotaurine increased. The results *in vitro* demonstrated that taurine significantly suppressed cellular proliferation, metastasis, and colony formation whereas it induced apoptosis in CRC cells. Furthermore, taurine regulated the expression levels of epithelial mesenchymal transition (EMT)-associated genes in a dose-dependent manner. Taurine also alleviated hypotaurine-induced CRC progression, which was linked to the inhibition of the ERK/RSK-signaling pathway and diminution in intracellular hypotaurine. Taurine additionally attenuated hypotaurine-induced tumor growth and metastasis *in vivo.* Patients with CRC exhibited lower levels of serum taurine, suggesting that taurine might be a promising biomarker reflecting a poor prognosis in CRC. Collectively, our results demonstrated that taurine-attenuated, hypotaurine-induced CRC progression provides a potential target for CRC therapy.

## Introduction

Colorectal cancer (CRC) is the third most common malignant disease worldwide, with an estimated 1.8 million new cases and 881,000 cancer deaths globally in 2018 (Bray et al., 2018; Siegel et al., 2019). The revised World Cancer Research Fund/American Institute for Cancer Research report points out that there is solid evidence that processed meat, alcohol, and obesity increase the risk of CRC. Although patients with early CRC can be cured by surgery (Shelton, 2002; Lieberman et al., 2012), patients with advanced CRC should not undergo surgery and chemotherapy (Imai et al., 2015). Therefore, more in-depth studies are needed regarding the underlying molecular mechanisms and drivers of CRC progression in order to find additional potential therapeutic targets and strategies.

Metabolomics can illuminate abnormal metabolic pathways associated with various tumor types. Taurine (2-aminoethanesulfonic acid) is a natural amino acid that is widely expressed in mammalian tissues, and is critical for maintaining the functioning of the central nervous system, retinal neurons, cardiac, and skeletal muscle (Schaffer et al., 2010; Gaucher et al., 2012; Kilb and Fukuda, 2017; Khalil et al., 2018). As an antipyretic and anti-inflammatory drug, taurine has been used in the treatment of diabetes, cataracts, and cardiovascular disease (Song et al., 2003; Son et al., 2007; Megaraj et al., 2010; Das et al., 2012; El Zahraa et al., 2012; Yin et al., 2012). Recent evidence also shows that taurine possesses anti-tumor properties in cancer (Zhang et al., 2014, 2015). For example, previous studies have shown that taurine, with diethylnitrosamine as carcinogen and phenobarbital as cancer promoter, exerts a protective effect on chemically induced hepatomas in male F344 rats (Okamoto et al., 1996). After taurine treatment of S180 transplanted tumors in nude mice, apoptosis was significantly increased (Wang, 2008). Taurine can also upregulate the expression of *N*-acetyl galactosaminyl transferase 2 and downregulate the expression of matrix metalloproteinase-2, thus inhibiting potential invasion and metastasis by glioma cells (Neary et al., 2010). However, studies of the effects on CRC of taurine and its metabolic precursor hypotaurine remain limited.

In the present study, we first performed gas chromatography-mass spectrometry (GC-MS) to analyze the metabolites in CRC tissues. This investigation revealed that taurine was downregulated, whereas hypotaurine was upregulated in CRC. Next, we determined the functions of taurine in the regulation of hypotaurine-induced tumor progression. Finally, we explored the detailed molecular mechanisms responsible for taurine’s function in CRC. Our data may provide new insights into the metabolic underpinnings of CRC progression, and offer key clues for developing new diagnostic and therapeutic targets for this devastating disease.

## Materials and Methods

### Patients and Clinical Samples

Forty-two CRC patients underwent radical surgery without preoperative anti-cancer therapy at Renji Hospital, Shanghai Jiao Tong University School of Medicine, were enrolled in this study. Our study was authorized by the Renji Hospital Ethics Committee, and all patients were required to sign written informed consent. Serum samples were obtained prior to treatment, and fresh tissue specimens were collected after surgery. All clinical samples were stored immediately after collection at −80°C.

### Cell Lines and Reagents

All human CRC cell lines were from the American Type Culture Collection (ATCC, Manassas, VA, United States) and authenticated by DNA profiling. All cells were cultured in DMEM supplemented with 10% FBS and 1% penicillin-streptomycin, in an incubator (Thermo Fisher Scientific, Waltham, MA, United States) at 37°C and 5% CO_2_ in air. A MycoSEQ Detection kit was used to detect mycoplasma every 6 months (4460626; Thermo Fisher Scientific). Cell lines were utilized for less than 6 months after receipt or resuscitation from cryopreservation. We purchased taurine (HY-B0351) and hypotaurine (HY-100803) from MedChemExpress (Monmouth Junction, NJ, United States). SCH772984 (S7101), Ro 318220 (S7207), and Stattic (S7024) were used to inhibit different ERK-signaling pathways (Selleck Chemicals, Houston, TX, United States).

### GC-MS and Liquid Chromatography-Tandem Mass Spectrometry (LC-MS/MS) in Metabolomics Analysis

Samples were prepared according to the manufacturer’s protocols (Human Metabolome Technologies, HMT, Tokyo, Japan). GC-MS was conducted using an Agilent 7890A gas chromatography system combined with an Agilent 5975C inert MSD system (Agilent Technologies, Santa Clara, CA, United States). We performed LC-MS/MS using an Agilent 6460 Triple Quad LC/MS system (Agilent Technologies) with an electrospray ionization (ESI) source and using positive multiple reaction monitoring (MRM), and applied Agilent Mass Hunter Ver.B.04.00 software (Agilent Technologies) to collect the MS data. Peak picking and metabolite determination were conducted according to the manufacturer’s protocol (HMT). Zero values were removed from the data based upon the 80%-rule as previously proposed (Bijlsma et al., 2006).

### *In vitro* Cell Viability Assay

Prior to the application of different reagents at the indicated final concentrations, 5,000 cells/well were plated in triplicate in 96-well plates. We determined viability through Alamar Blue analysis as described after 5–7 days of culture (Zhang et al., 2017). All samples were tested in triplicate.

### Soft-Agar Assay

Cells (5,000–10,000) were suspended in 0.3% agar containing DMEM and 10% FBS, and plated in triplicate in 24-well plates. After 3–4 weeks, the number of colonies per well was manually counted under an anatomical microscope (Olympus, Tokyo, Japan).

### Caspase 3/7 Reporter Assay

All cells were seeded in triplicate in 96-well plates, and treated with different reagents for 24 h. The data were analyzed using a Synergy H4 Hybrid Multimode Microplate Reader (BioTek, Winooski, VT, United States). We purchased a caspase 3/7 reporter kit (G8091) from Promega (Madison, WI, United States).

### Wound-Healing Assay

A total of approximately 2 × 10^5^ cells were plated in 6-well plates. After starving overnight in the medium supplemented with 1% FBS, a confluent monolayer of >90% was scraped with a 200 μl pipette tip to form a linear wound. The plates were washed with PBS and incubated in complete medium with or without reagents for 24 h. A phase-contrast optical microscope (Olympus) was used to capture the wound images, and the horizontal distances between the edges of the wounds were measured. All sampling was conducted in triplicate.

### Transwell Invasion Assay

A total of approximately 5 × 10^4^ cells were suspended in 200 μl of DMEM without serum and plated on the upper chamber of transwell filters in 24-well plates coated with Matrigel (354234; BD Biosciences, Franklin Lakes, NJ, United States), and filled with 500 μl of DMEM and 10% FBS with or without experimental agents. The lower chamber was fixed with 600 μl of DMEM. After 36 h of culture at 37°C, the non-invasive cells on the upper side of the membrane were removed, and the cells on the lower side of the membrane were fixed with 100% methanol for 20 min and stained with 1% crystal violet for 15 min at 37°C. The cell images were collected and five random fields were counted under an anatomical microscope. The sampling was conducted in triplicate.

### Quantitative Real-Time Reverse Transcription Polymerase Chain Reaction Analysis

Trizol reagent (15596018; Thermo Fisher Scientific) was used to isolate total RNA from cells or tissues, and a One-Step RT-PCR kit (Qiagen, Dusseldorf, Germany) was used to synthesize cDNA. The ABI 7500 Real-time PCR system (Thermo Fisher Scientific) was used to evaluate the expression of CDH1, Snail, and FMO1. The primers were as follows: CDH1, 5′- AAGTGCTGCAGCCAAAGACAGA-3′ (forward) and 5′-AA ATTGCCAGGCTCAATGACAAG-3′ (reverse); Snail, 5′-GGA AGCCTAACTACAGCGAGC-3′ (forward) and 5′-AGGACAGA GTCCCAGATGAGC-3′ (reverse); FMO1, 5′-GAGCGAAAGA TAAACAACTGGCT-3′ (forward) and 5′-TGCTTGGCCTGAT GAACACTT-3′ (reverse); GAPDH, 5′-GCACCGTCAAGGCT GAGAAC-3′ (forward); and 5′-ATGGTGGTGAAGACGCCA GT-3′ (reverse). GAPDH was used as a reference and the 2^–ΔΔ*Ct*^ methods were applied to quantify the relative expression levels.

### Western Blotting Analysis

After treatment, RIPA lysis buffer [25 mmol/L Tris (pH7.4), 150 mmol/L NaCl, 5 mmol/L EDTA, and 1% Triton-X] plus phosphatase and protease inhibitors were used to harvest cells. A total of 30–50 μg of lysate was dissolved in the SDS-PAGE gels and then transferred to PVDF membranes (1620177; Bio-Rad, Hercules, CA, United States) that were blocked. The identification of target proteins was conducted with unique antibodies, followed by secondary antibodies. The commercial antibodies were purchased from Cell Signaling Technology (CST, Boston, MA, United States) as follows: rabbit monoclonal antibody to ERK1/2 (1:1000, 4695), rabbit monoclonal antibody to RSK (1:1000, 9355), phospho-ERK1/2 (T202/204) antibody (1:1000, 4370), phospho-RSK (S380) antibody (1:1000, 11989), and mouse monoclonal antibody to β-actin (1:1000, 3700). The secondary antibodies used in the experiments were IRDye 680RD donkey anti-mouse IgG (1:10000, 925-68072; LI-COR Biosciences, Lincoln, NE, United States) and IRDye 800CW goat anti-rabbit IgG (1:10000, 926-32211; LI-COR Biosciences). The blots were scanned using an Odyssey infrared imaging system (LI-COR Biosciences).

### Xenograft Tumor Model

Our protocol was approved by the Ethics Committee of Renji Hospital. A total of 5 × 10^6^ CRC cells were subcutaneously injected into the athymic flanks of 4-week-old BALB/c nude mice (Shanghai Laboratory Animal Center, Shanghai, China). Treatment was initiated when tumors approached a volume of approximately 100 mm^3^, and tumor volume was calculated once per week using the formula V = 1/2 (L × W^2^) (where L = length and W = width). The mice were then allocated to four groups according to the 4-week treatments as follows: (a) saline (vehicle), (b) taurine at 200 mg/kg/day, (c) hypotaurine at 200 mg/kg/day, and (d) taurine at 200 mg/kg/day plus hypotaurine at 200 mg/kg/day. At the end of the study, mice were euthanized, tumor weights were recorded, and the tumor tissues were collected for further analysis.

### Orthotopic Model

Six-week-old NOD SCIDγ (NSG) mice were purchased from the Shanghai Laboratory Animal Center. The median incision in the abdominal wall was 1 cm, and the cecum was externalized. The mucous membrane was weakened in advance by mild rubbing against the wall to promote implantation, which was necessary to prevent tumor cells from infiltrating the cecal or peritoneal cavities. A 100 μl suspension containing 5 × 10^6^ CT26 cells was then injected into the cecal wall. Mice were divided into four groups (six mice per group) seven days after implantation. We measured bioluminescence imaging (BLI) signals using the PhotonIMAGER Optima System (Biospace Lab, Nesles la Vallée, France), which can detect the presence of cecal, hepatic, and pulmonary tumors or micrometastases. Twenty-five days after implantation, the cecums, livers, and lungs were collected from treated mice and controls, and organs were fixed in 4% paraformaldehyde for 4 h for subsequent histologic and immunohistochemical analyses.

### Histologic, Immunohistochemical, and Immunofluorescence Analyses

Tissues were stained with H&E after resection, fixed, and paraffin-embedded. Tumor nodules in livers and lungs were selected to evaluate the level of metastasis. For immunohistochemistry, 4 μm sections of tissue were incubated with cytokeratin 19 (1:200, 4,558; CST) antibody, and for immunofluorescence, fixed sections were incubated with primary antibodies to Ki67 (1:50, 9,449; CST), pan-keratin (1:50, 4,545; CST), or cleaved-caspase 3 (1:200, 9,664; CST). We quantified the number of Ki67-positive cells and apoptotic areas from two independent tumors of each group; this entailed 10 sections/tumor and 1 field/section, using a 20× objective, NIS-Elements software (Nikon, Tokyo, Japan), and a C2 + fluorescence confocal microscope.

### Statistical Analysis

The data are presented as means ± standard error of the mean (SEM). We used an unpaired Student’s *t* test to compare two groups, and one-way ANOVA followed by Tukey’s *post hoc* test for multiple comparisons. The Prism 6 software program was used to analyze the data. All tests were two-tailed, and *P* < 0.05 was considered significant.

## Results

### GC-MS of Metabolic Changes in CRC Tissues

**FIGURE 1:**
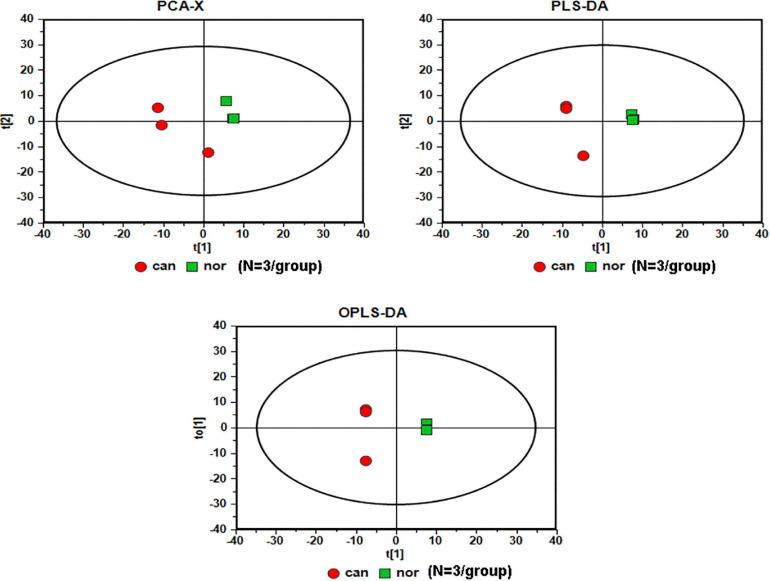
Serummetabolic profiles derived from CRC tissues are different from those of healthy subjects (*N* = 3/group). Red circles, cancer samples; green squares, normal samples.

We used GC-MS to examine the alterations of metabolites in CRC. We included World Health Organization (WHO) grade IV (*n* = 3) CRC specimens, and the normal colonic tissue around the tumor obtained during the operation was used as the control sample (*n* = 3). The analysis of raw GC-MS data has been described in previous protocols (Gao et al., 2010). The peak table file was imported into SIMCA (Version 11.0, Umetrics, Umea, Sweden), including principal component analysis (PCA), partial least squares-discriminant analysis (PLS-DA), and orthogonal partial least squares-discriminant analysis (OPLS-DA). Two PCs were each measured for tissue extracts explained by t1 and t2. The score curve showed that each group was scattered in different areas that represented a significant separation of cancer and non-cancer samples, indicating that colon cancer tissue had a specific metabolic spectrum that was different from that of the controls (Figure 1).

### Taurine and Hypotaurine in CRC Tissues

**FIGURE 2:**
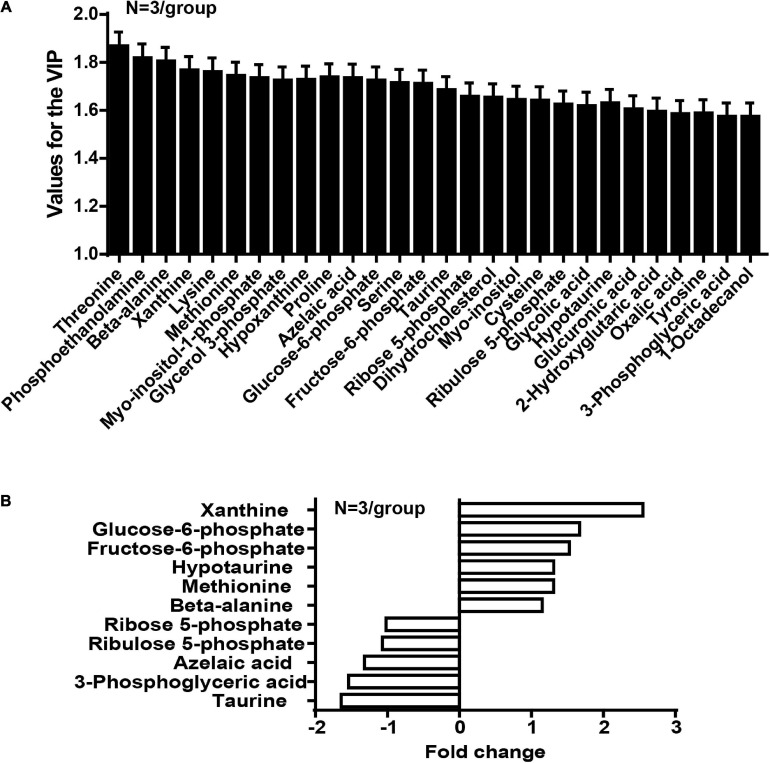
The concentration of taurine is diminished whereas the concentration of hypotaurine is augmented in CRC tissues (*N* = 3/group). **(A)** Values for the VIP of 28 characteristics are depicted. Each column displays one feature of the PLS-DA model (Figure 1). Error bars represent the SEM. **(B)** Different levels of compounds between cancer and normal groups (2-tailed *t* test, *P* < 0.05). The y-axis represents the various compounds, and the x-axis represents fold-change in metabolite levels.

In the OPLS-DA model, the variable importance in the project (VIP) was employed to express the importance of different features to sample discrimination. Characteristics with VIP values greater than one were chosen for further consideration, including the development of discriminatory models and the recognition of chemical structures. We analyzed and queried the parameters of each feature in the following databases: Kyoto Encyclopedia of Genes and Genomes (KEGG^1^), Human Metabolome Database (HMDB^2^), and Mass Bank^3^. The fold-change was measured as the binary logarithm of the mean normalized peak-intensity ratio between the cancer and normal groups, and a positive value indicated that the mean quality response of the cancer group was greater than that of the normal group. We ultimately identified the structures of 28 metabolites, and observed that the content of taurine in the cancer group was lower than that in the normal group, and that the content of hypotaurine in the cancer group was reciprocally higher than in the normal group (Figure 2).

### Taurine Attenuates Hypotaurine-Induced Tumor Progression in CRC Cells

**FIGURE 3:**
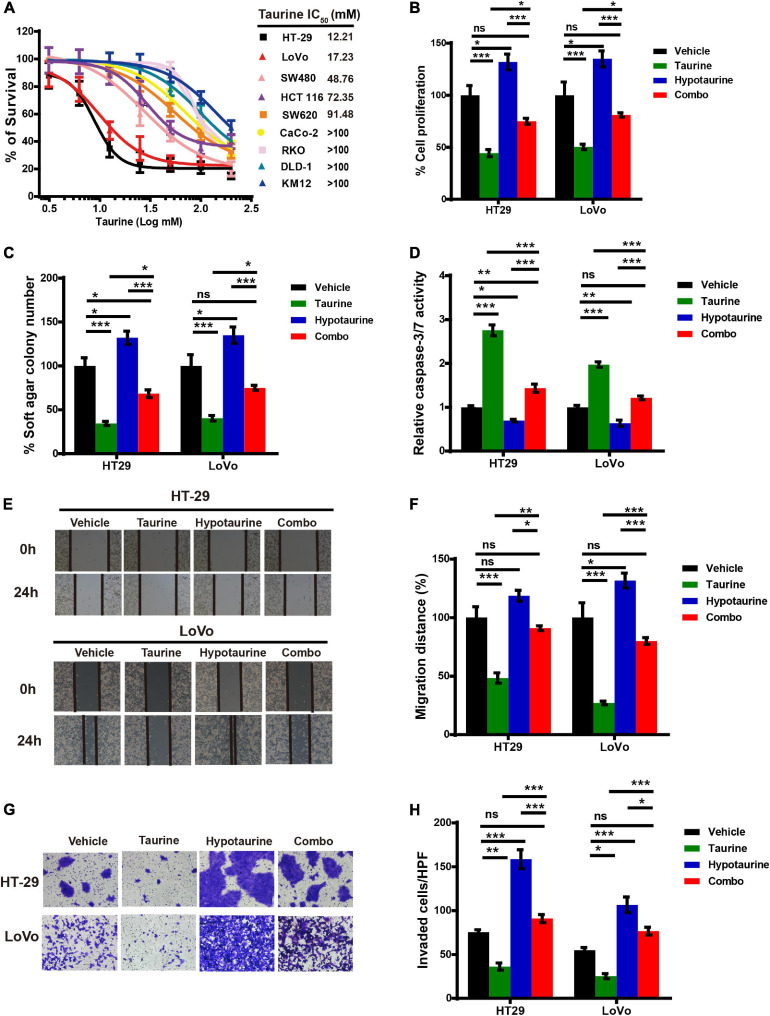
Taurine reverses the promoting effects of hypotaurine on CRC progression. **(A)** The viability of CRC cell lines cultured for five days in serial concentrations of taurine, and each IC50 value were shown by Alamar Blue assay (means ± SEM). **(B)** The proliferative rate of CRC cell lines after treatment with taurine (10 mM) and/or hypotaurine (10 mM) (means ± SEM) (one-way ANOVA followed by Tukey’s *post hoc* test, *P < 0.05, ***P < 0.001). **(C)** Quantification of clones formed by the indicated CRC lines after treatment with taurine (5 mM) and/or hypotaurine (5 mM) for 3 weeks (means ± SEM) (one-way ANOVA followed by Tukey’s *post hoc* test, *P < 0.05, ***P < 0.001). **(D)** The proapoptotic effects of taurine (10 mM) and/or hypotaurine (10 mM) on CRC cells were detected by caspase 3/7 reporter assay (means ± SEM) (one-way ANOVA followed by Tukey’s *post hoc* test, *P < 0.05, **P < 0.01, ***P < 0.001). **(E,F)** The cellular migratory effects of taurine (10 mM) and/or hypotaurine (10 mM) in CRC cells were evaluated by wound-healing assay and quantified. (means ± SEM) (one-way ANOVA followed by Tukey’s post- hoc test, *P < 0.05, ***P < 0.001). **(G,H)** The cellular invasive effects of taurine (10 mM) and/or hypotaurine (10 mM) in CRC cells were assessed using the transwell invasion assay and quantified (means ± SEM) (one-way ANOVA followed by Tukey’s *post hoc* test, *P < 0.05, **P < 0.01, ***P < 0.001).

We first examined the effect of taurine as a single agent in nine conventional monolayer-cultured CRC cell lines. After five days of culture in a 2D condition, the cell viability of all CRC cell lines was inhibited in a concentration-dependent manner. HT-29 and LoVo were two representative CRC cell lines that showed a significant difference in taurine sensitivity, and were therefore chosen for subsequent studies (Figure 3A). A variety of *in vitro* assays were employed to assess the effects of taurine on cellular functions, including proliferation, apoptosis, migration, and invasion, in order to identify whether taurine was functionally involved in CRC cells. Figure 3B illustrates that upregulation of taurine inhibited the proliferation of HT-29 and LoVo cells through Alamar Blue assays (Figure 3B), and colony formation assays verified that taurine exerted an inhibitory action on cellular proliferation (Figure 3C). Caspase-3/7 activity was measured to investigate the apoptotic action of taurine on both cell lines, and it significantly increased after treatment with taurine for 24 h (Figure 3D). In the wound-healing migration assay, taurine-treated cells displayed a suspension of wound healing (Figures 3E,F); and transwell assays showed that taurine significantly impaired cellular invasion in both cell lines (Figures 3G,H). To determine whether elevated taurine played an important role in hypotaurine induced-CRC progression, we treated CRC cells with a combination of taurine and hypotaurine, and the results revealed that taurine rescued the suppression of apoptosis, and enhanced proliferation, migration, and invasion of hypotaurine-treated CRC cells (Figures 3A–H).

### Taurine Attenuates the Hypotaurine-Induced Epithelial Mesenchymal Transition in CRC Cells

**FIGURE 4:**
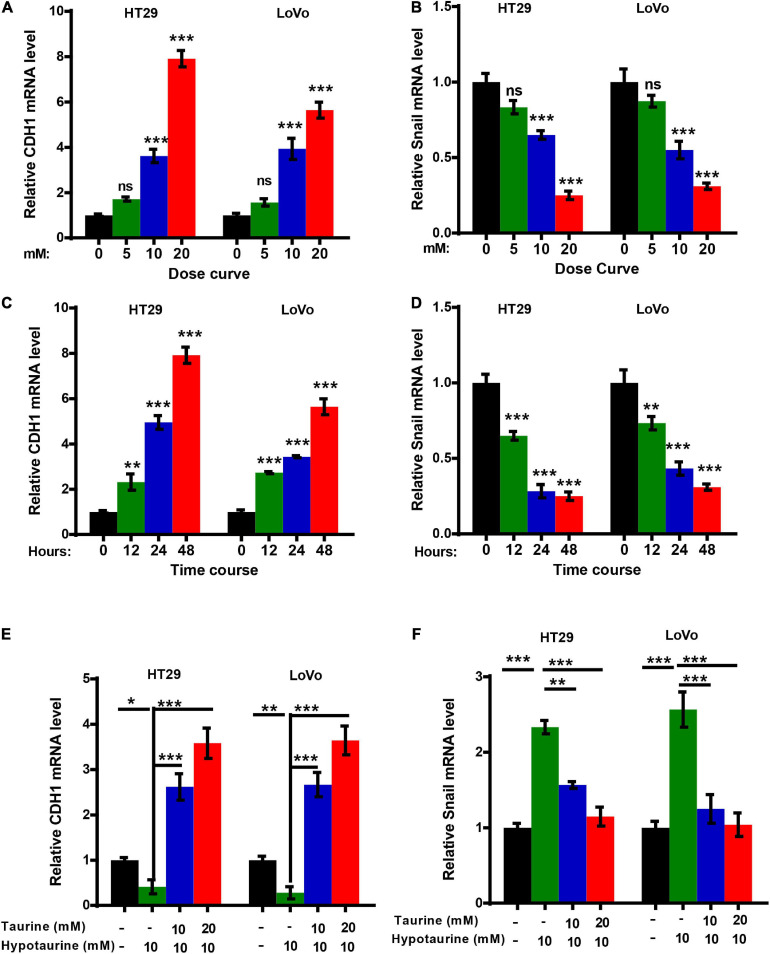
Taurine reverses the promoting effect of hypotaurine on the EMT in CRC cells. **(A,B)** The dose-effect curve for taurine on EMT related-gene expression in CRC cells was determined by qRT-PCR (means ± SEM) (one-way ANOVA followed by Tukey’s *post hoc* test, ***P < 0.001). **(C,D)** The time-effect curve for taurine on EMT related-gene expression in CRC cells was developed using qRT-PCR (means ± SEM) (one-way ANOVA followed by Tukey’s *post hoc* test, **P < 0.01, ***P < 0.001). **(E,F)** The effects of taurine and/or hypotaurine on EMT markers in CRC cells were measured by qRT-PCR (means ± SEM) (one-way ANOVA followed by Tukey’s *post hoc* test, *P < 0.05, **P < 0.01, ***P < 0.001).

The epithelial mesenchymal transition (EMT) is one of the critical factors in cellular proliferation, apoptosis, and metastasis. We determined the change in EMT markers in CRC cells with quantitative real-time reverse transcription polymerase chain reaction (qRT-PCR) analysis, and showed upregulation of CDH1 expression and downregulation of Snail in HT-29 and LoVo cells with taurine treatment. Compared with the control group, these effects persisted with increasing dose and duration of taurine treatment (Figures 4A–D), and thus taurine affected CDH1 and Snail expression levels in a dose- and time-dependent manner. Our data also showed that taurine reversed hypotaurine-mediated expression of EMT markers in CRC cells (Figures 4E,F).

### Taurine Attenuates Hypotaurine-Induced Tumor Progression and the EMT *via* the Extracellular-Signal Regulated Kinase (ERK)/Ribosomal S6 Kinase (RSK) Pathway, and Decreases Hypotaurine Level in CRC Cells

**FIGURE 5:**
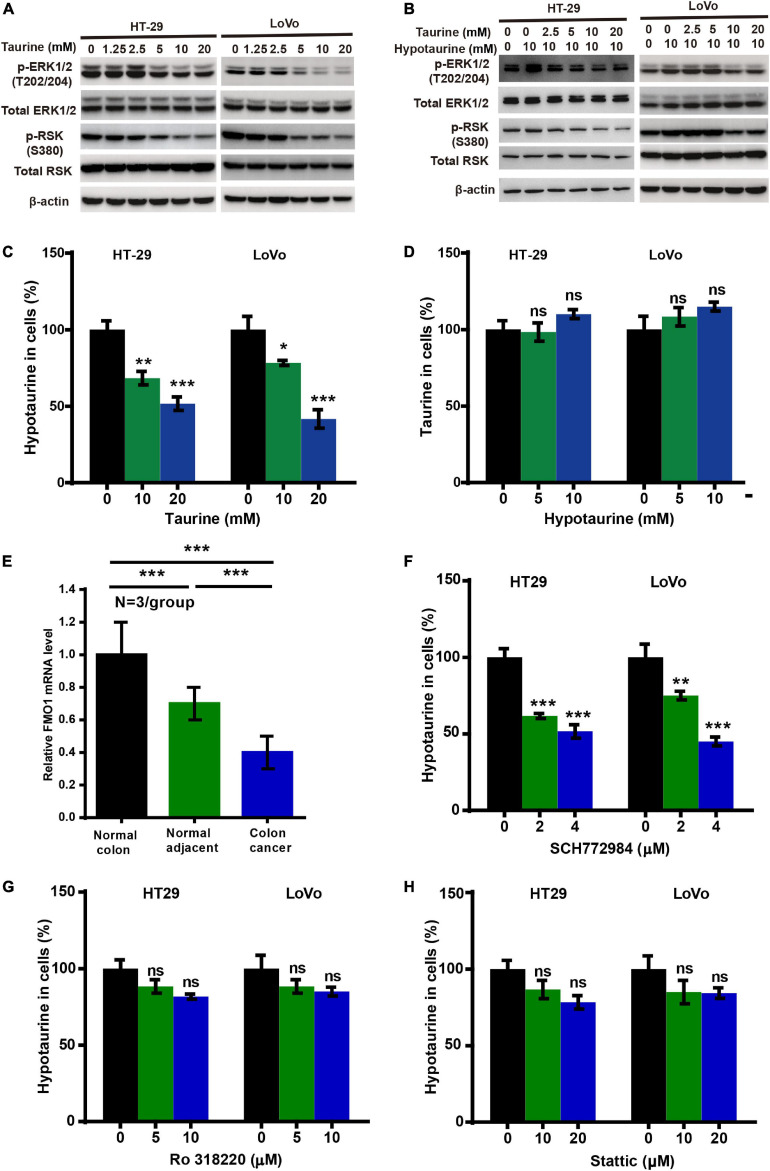
Taurine reverses the promoting effects of hypotaurine on tumor progression and EMT through the ERK/RSK pathway, and decreases intracellular hypotaurine levels in CRC cells. **(A,B)** The effects of taurine and/or hypotaurine on the phosphorylation (denoted p-) status of ERK and RSK were assessed with western blotting analysis. **(C,D)** CRC cells were incubated for 24 h in medium with the indicated concentrations of taurine or hypotaurine, and intracellular hypotaurine or taurine concentrations were identified by LC-MS analysis (means ± SEM) (one-way ANOVA followed by Tukey’s *post hoc* test, *P < 0.05, **P < 0.01, ***P < 0.001). **(E)** FMO1 mRNA expression in normal colon, normal colonic tissues adjacent to cancer, and colon cancer were determined by qRT-PCR (means ± SEM) (*N* = 3/group; one-way ANOVA followed by Tukey’s post- hoc test, ***P < 0.001). **(F–H)** CRC cells were incubated for 24 h in medium with the indicated concentrations of different ERK inhibitors (SCH772984, Ro 318220, and Stattic), and intracellular hypotaurine concentrations were identified by LC-MS analysis (means ± SEM) (one-way ANOVA followed by Tukey’s *post hoc* test, **P < 0.01, ***P < 0.001).

To investigate the possible mechanism underlying taurine inhibition of hypotaurine-induced progression and EMT in CRC cells, we analyzed taurine and/or hypotaurine effects on the ERK pathway through western blotting analysis. Taurine significantly suppressed the levels of p-ERK and p-RSK in CRC cells, while hypotaurine induced ERK activation (Figures 5A,B). These data suggested that the ERK/RSK pathway may participate in taurine regulation of hypotaurine-induced progression and the EMT of CRC. Hypotaurine concentrations decreased significantly in a dose- dependent manner over a 24-h taurine exposure, while taurine levels did not increase during prolonged incubation of cells with these precursors (Figures 5C,D). This suggested that the enzymatic conversion of hypotaurine to taurine was either lacking or strictly regulated in these cell lines. The investigators recently demonstrated that the flavin-containing monooxygenase 1 (FMO1) oxygenated hypotaurine to produce taurine both *in vivo* and *in vitro* (Veeravalli et al., 2020). We thus executed qRT-PCR analysis using colonic tissues, and found the level of FMO1 mRNA to be significantly decreased in CRC compared to normal colonic tissues (Figure 5E). It has been reported that the phosphorylation-activated ERK1/2 translocates to the nucleus where it phosphorylates and activates mitogen- and stress-activated protein kinase (MSK), RSK, and signal transducer and activator of transcription 3 (STAT3) to stimulate cellular growth and proliferation (Zhang and Liu, 2002). To explore the possible downstream effectors of ERK1/2 signaling (besides RSK) in CRC cells, we analyzed the effects of three different ERK inhibitors on hypotaurine levels by LC-MS analysis. The results suggested that only the ERK/RSK inhibitor could regulate intracellular hypotaurine levels (Figures 5F–H). Collectively, our results strongly support our contention that the ERK/RSK pathway and decreased hypotaurine levels play important roles in taurine-mediated inhibition of tumor progression and the EMT.

### The Inhibition of ERK/RSK Signaling Mimics the Counteracting Effects of Taurine on Hypotaurine

**FIGURE 6:**
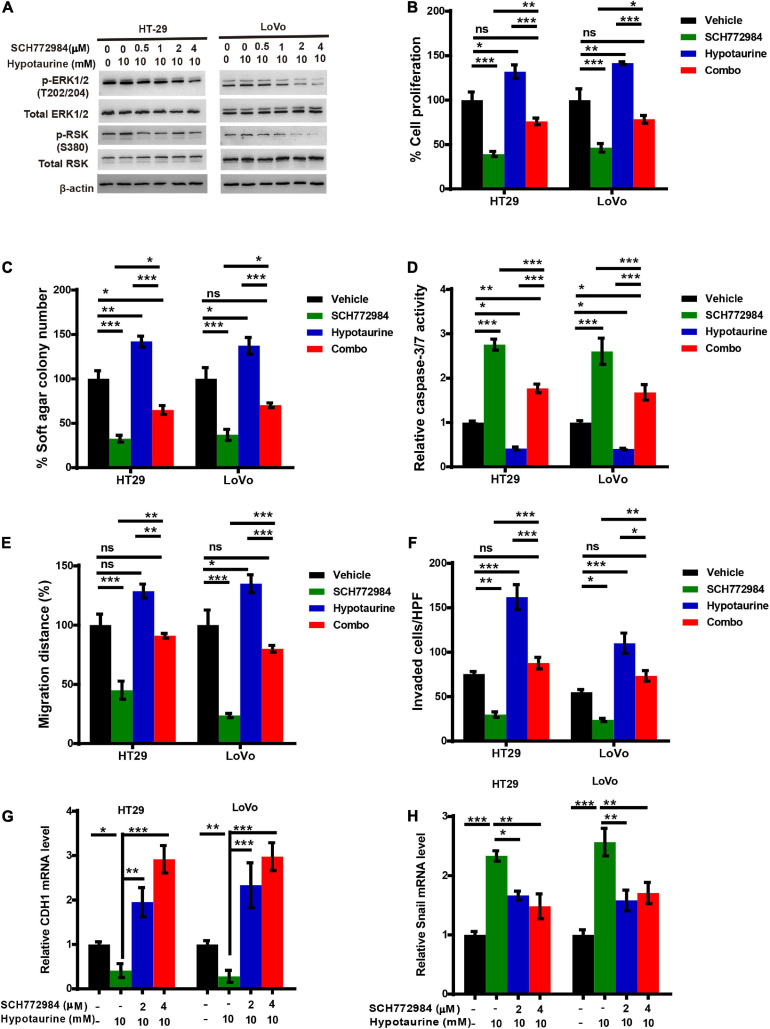
An ERK/RSK inhibitor reverses the promoting effects of hypotaurine on tumor progression and EMT in CRC cells. **(A)** The effects of ERK/RSK inhibitor (SCH772984) on the phosphorylation (denoted p-) status of ERK and RSK were detected with western blotting analysis. **(B)** The proliferative rates of CRC cell lines after treatment with SCH772984 (2 μM) and/or hypotaurine (10 mM) (means ± SEM) (one-way ANOVA followed by Tukey’s *post hoc* test, *P < 0.05, **P < 0.01, ***P < 0.001). **(C)** Quantification of clones formed by the indicated CRC lines after treatment with SCH772984 (1 μM) and/or hypotaurine (5 mM) for 3 weeks (means ± SEM) (one-way ANOVA followed by Tukey’s *post hoc* test, *P < 0.05, **P < 0.01, ***P < 0.001). **(D)** The proapoptotic effects of SCH772984 (2 μM) and/or hypotaurine (10 mM) on CRC cells were observed with caspase 3/7 reporter assay (means ± SEM) (one-way ANOVA followed by Tukey’s *post hoc* test, *P < 0.05, **P < 0.01, ***P < 0.001). **(E)** The cellular migratory effects of SCH772984 (2 μM) and/or hypotaurine (10 mM) on CRC cells were determined by wound-healing assay and quantified. (means ± SEM) (one-way ANOVA followed by Tukey’s *post hoc* test, *P < 0.05, **P < 0.01, ***P < 0.001). **(F)** The cellular invasive effects of SCH772984 (2 μM) and/or hypotaurine (10 mM) on CRC cells were ascertained by transwell invasion assay and quantified (means ± SEM) (one-way ANOVA followed by Tukey’s *post hoc* test, *P < 0.05, **P < 0.01, ***P < 0.001). **(G,H)** The effects of SCH772984 and/or hypotaurine on EMT markers in CRC cells were discerned using qRT-PCR (means ± SEM) (one-way ANOVA followed by Tukey’s *post hoc* test, *P < 0.05, **P < 0.01, ***P < 0.001).

Previous research has established that RSKs are important regulators of migration and invasion in response to activation of the ERK/MAPK-signaling pathway (Sulzmaier and Ramos, 2013). To further verify whether ERK/RSK signaling contributed to hypotaurine-induced CRC progression and the EMT, we treated CRC cells with a combination of the ERK/RSK inhibitor and hypotaurine, and showed that SCH772984 rescued suppressed apoptosis and enhanced proliferation, migration, and invasion of hypotaurine-treated CRC cells (Figures 6A–F and Supplementary Figures 7, 8). Furthermore, our data showed that SCH772984 reversed hypotaurine-mediated expression of EMT markers in CRC cells (Figures 6G,H). Taken together, our results strongly support our hypothesis that the inhibition of ERK/RSK signaling mimics the preventive effects of taurine on hypotaurine.

### Taurine Attenuates Hypotaurine-Induced Tumor Growth *in vivo*

**FIGURE 7:**
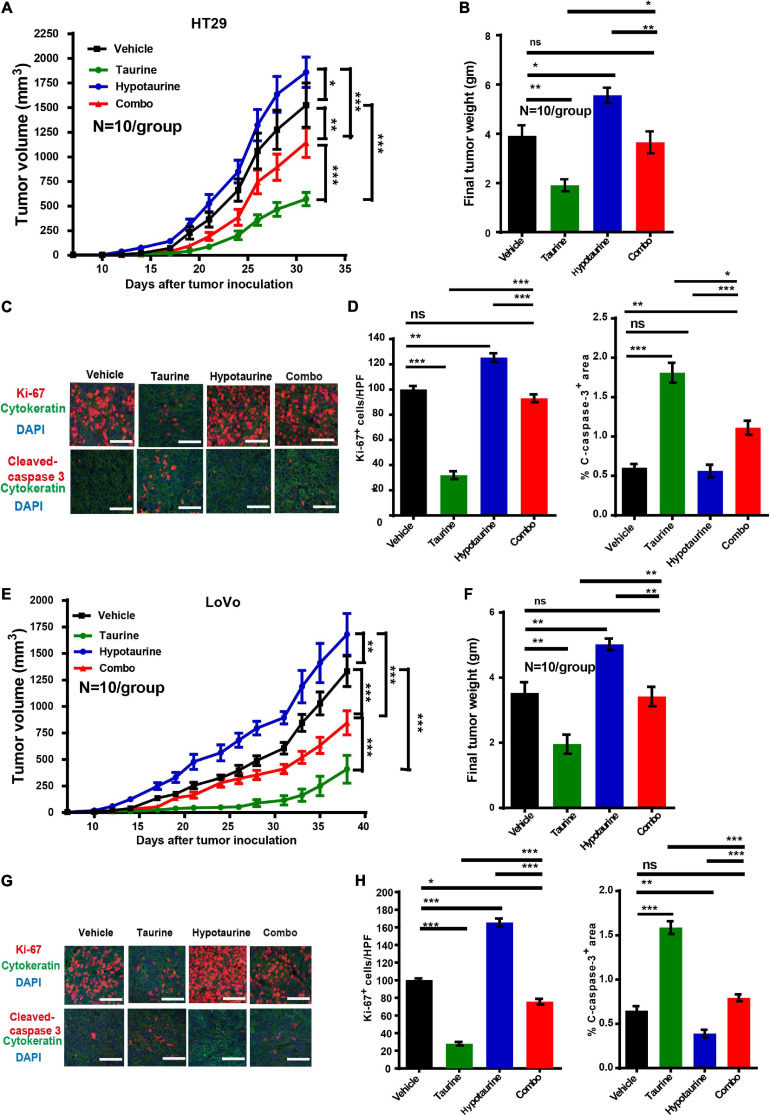
Taurine reverses the promoting effects of hypotaurine on tumor growth *in vivo*. **(A,B)** Serial measurements of tumor volume and final weights of HT-29 tumors grown subcutaneously in nude mice treated as indicated (means ± SEM) (*N* = 10/group; one-way ANOVA followed by Tukey’s *post hoc* test, *P < 0.05, **P < 0.01, ***P < 0.001). **(C,D)** Representative photomicrographs **(C)** and quantification of cleaved caspase-3^+^ area per × 200 field and dual CK^+^ and Ki-67^+^ cells per × 400 field **(D)** of HT-29 tumors treated as indicated using immunofluorescence. Ten random photographs were taken from each of 10 tumors per group (mean ± SEM) (one-way ANOVA followed by Tukey’s *post hoc* test, **P < 0.01, ***P < 0.001; scale bars, 50 μm). **(E,F)** Serial measurements of tumor volume and final weights of LoVo tumors grown subcutaneously in nude mice treated as indicated (means ± SEM) (*N* = 10/group; one-way ANOVA followed by Tukey’s post- hoc test, **P < 0.01, ***P < 0.001). **(G,H)** Representative photomicrographs **(G)** and quantification of cleaved caspase-3^+^ area/200 field, and dual CK^+^ and Ki-67^+^ cells/400 field **(H)** of LoVo tumors treated as indicated and determined by immunofluorescence. Ten photomicrographic images were captured randomly from each of 10 tumors per group (mean ± SEM) (one-way ANOVA followed by Tukey’s *post hoc* test, *P < 0.05, **P < 0.01, ***P < 0.001; scale bars, 50 μm).

Our research has shown that taurine participates in hypotaurine-induced CRC growth *in vitro*, and that taurine reverses tumor growth in hypotaurine-treated xenograft mouse models. HT-29 and LoVo cells readily form tumors in immunocompromised mice, with a short latency period (∼14 days), and these were established in our nude mice (∼100 mm^3^). Hypotaurine alone promoted tumor growth, but combination-treated tumors were lower in tumor volume and weight in both models (Figures 7A,B,E,F). Compared with hypotaurine treatment, the combination-treated tumors displayed increased apoptosis (as assessed by cleaved-caspase-3^+^ area) and decreased proliferation (Ki-67^+^) of neoplastic cells (dual pan-CK^+^Ki-67^+^cells) (Figures 7C,D,G,H). Our collective results strongly support taurine as reversing hypotaurine-induced tumor growth.

### Taurine Attenuates Hypotaurine-Mediated Tumor Metastasis *in vivo*

**FIGURE 8:**
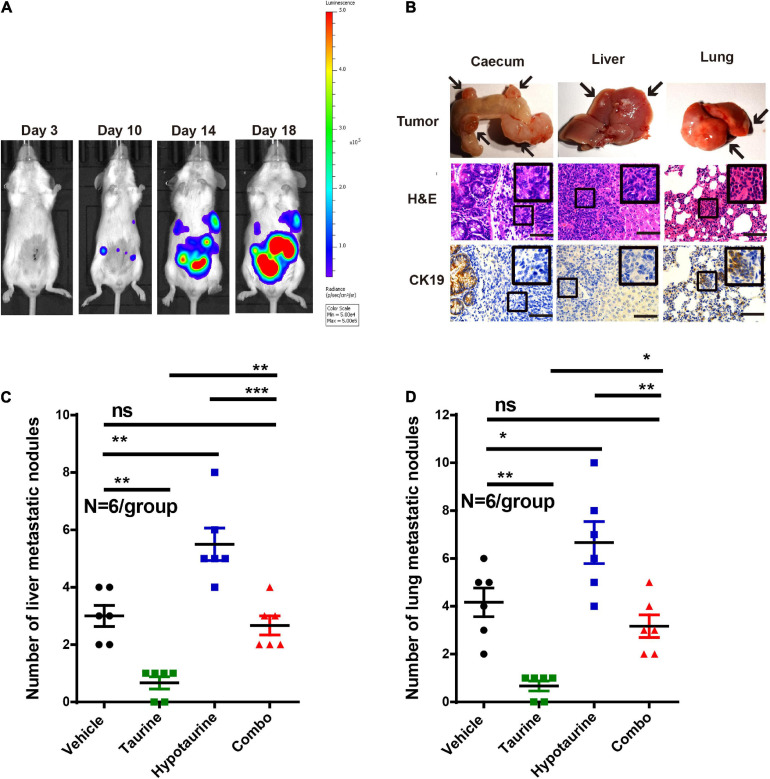
Taurine reverses the promoting effects of hypotaurine on tumor metastasis *in vivo*. **(A)** Representative images of total mouse luciferase signal were shown, manifesting the presence of tumor formation, liver, and lung metastases. **(B)** The cecum, liver, and lung of mice were stained with H&E and cytokeratin 19. The arrows represented macroscopic cecal tumor and metastatic spots (magnification 200×; scale bars, 50 μm). **(C,D)** Quantitative results of metastatic area as liver/lung (mean ± SEM) (*N* = 6/group; one-way ANOVA followed by Tukey’s *post hoc* test, *P < 0.05, **P < 0.01, ***P < 0.001).

As taurine was shown to attenuate the migration and invasion of hypotaurine-treated CRC cells, its effects *in vivo* were further examined. We thereby established an orthotopic colonic tumor model using spontaneous lung and liver metastasis xenografts in BALB/c mice. We initiated treatment once tumor establishment was confirmed by a positive bioluminescence signal. On day 25 (the day of sacrifice), macroscopic pulmonary and hepatic metastases were determined by bioluminescence signals and spots of tissue necrosis on the organs. H&E and cytokeratin 19 staining of lung and liver tissues were performed to assess tumor cell metastasis. Our representative photomicrographs depict the metastases of tumor cells to liver and lung, and the formation of tumor nodules (Figures 8A,B). Although the number of metastatic nodules in livers and lungs decreased with taurine treatment, hypotaurine promoted CRC cell metastasis (Figures 8C,D) indicating that taurine could suppress the metastasis of hypotaurine-induced colonic cancer to liver and lung.

### Low Levels of Taurine in Serum Are Associated With a Poor Prognosis in CRC Patients

**FIGURE 9:**
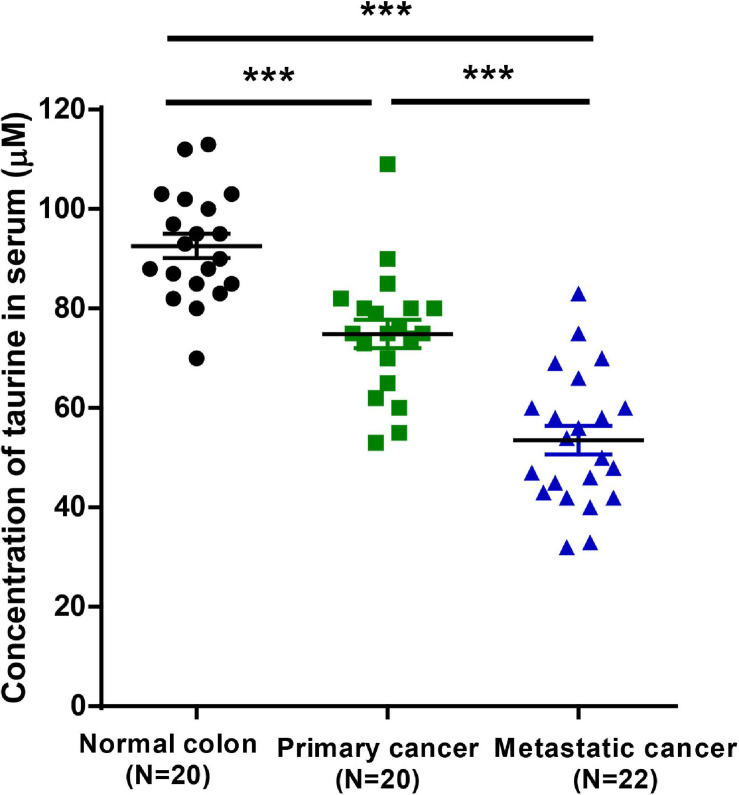
Downregulated taurine levels in serum are correlated with a poor prognosis in CRC patients (mean ± SEM) (*N* = 20 normal and 42 patients; one-way ANOVA followed by Tukey’s *post hoc* test, ***P < 0.001).

To investigate whether taurine might serve as a potential prognostic biomarker for CRC patients, we measured serum taurine levels in healthy donors and CRC patients by LC-MS. It is worth noting that when other clinicopathologic factors were controlled (Supplementary Table 1), we found that patients with metastatic CRC exhibited significantly lower serum concentrations of taurine relative to patients with primary CRC (Figure 9). These results thus showed that low serum taurine was correlated with poor prognosis in CRC patients.

## Discussion

The purpose of our study was to identify the abnormal metabolic pathways in CRC and their effects on tumor progression. Using GC-MS, we identified 28 metabolites, and also noted lower taurine and higher hypotaurine levels in the cancer group compared to the normal group. However, the biologic roles of taurine and hypotaurine remain elusive. The biosynthesis of taurine includes the oxidation of cysteine to cysteinesulfinic acid as catalyzed by cysteine dioxygenase, decarboxylation catalyzed by cysteine sulfite decarboxylase, and the oxidation of hypotaurine to taurine by hypotaurine dehydrogenase (Vitvitsky et al., 2011). In our study, taurine and hypotaurine might therefore play a critical role in the mechanisms underlying CRC. Although promising progress has been made in early diagnosis, surgical techniques, and chemotherapy, the prognosis of patients with CRC remains disappointing. Because abnormal metabolomic profiles have been reported to be linked to tumor progression (Chinnaiyan et al., 2012; Hadi et al., 2017), the elucidation of novel CRC-associated metabolomic biomarkers is critical for early diagnosis and to provide an effective strategy for advanced CRC.

A number of investigators have demonstrated that the antitumor effects of taurine contribute to its ability of promoting apoptosis and reducing proliferation (He et al., 2018; Tu et al., 2018). Gao et al. (2016) reported an enhancement of glioma cell proliferation and invasiveness by hypotaurine. In our study, taurine was examined as a single agent using a panel of CRC cell lines, and we observed a concentration-dependent inhibition of cellular viability in all the lines with a five day culture in 2D condition. HT-29 and LoVo were selected for subsequent investigation of the differences in their sensitivities to taurine, and a series of experiments was conducted to determine the effects of taurine on the development of CRC. Taurine induced apoptosis, inhibited cellular proliferation, migratory and invasive capabilities. We then evaluated CDH1 and Snail proteins as crucial markers of the EMT, as the EMT is principally involved in cellular proliferation, apoptosis, and metastasis. We found that taurine treatment promoted CDH1 levels while it inhibited Snail levels, indicating that taurine influences CRC cell metastasis, apoptosis, and proliferation involved in the EMT. We postulate that our results will engender a better understanding of the effects of taurine as a mediator of CRC pathophysiology, and the results indicate that taurine attenuates the malignant phenotype evoked by hypotaurine. The inhibitory effect of taurine on CRC thus suggests that taurine treatment may constitute a promising solution to halting the progression of CRC.

The mechanisms associated with taurine-mediated inhibition of the EMT in CRC are currently arcane. The proteins in the ERK-signaling pathway appear hyperactive and overexpressed in various forms of cancer, including CRC (Lok et al., 2011; Blaj et al., 2017), and the ERK pathway is known to regulate the EMT, tumor cell migration, invasion, and cell-cycle progression (Olea-Flores et al., 2019). Because of the specific structural properties of ERK, its mechanism of action, feedback mediation, and the various substrates catalyzed, ERK1/2 is a potential candidate for the development of strategies that could be used to inhibit tumor progression. However, the development of ERK-pathway molecules as therapeutic targets remains challenging because the potential for off-targeting and toxicity of these new compounds has also increased. It should be noted, however, that taurine as a natural amino acid can overcome these problems. Our *in vitro* results showed that phosphorylated ERK1/2 and RSK expression were reduced with taurine treatment in a dose-dependent manner, and with increasing hypotaurine concentration, ERK1/2 and RSK phosphorylation also increased commensurately. This dose-dependent activity may represent the primary mechanism by which taurine and hypotaurine exert opposing effects on CRC cells.

Additionally, our experiments showed an aberrant metabolic pathway involved in taurine-inhibited tumor progression in CRC cells. Taurine reduced intracellular hypotaurine levels, indicating that the availability of extracellular taurine may diminish synthesis of *de novo* hypotaurine. Previous evidence has shown decreased taurine levels in glioma tissue, and we found that alterations in taurine levels changed intracellular hypotaurine levels (Gao et al., 2016). In these cell lines, either the enzymatic conversion of subtaurine to taurine was lacking, or the transformation was strictly regulated; this inhibition might then be involved in the mechanism subserving reduced hypotaurine levels. We hypothesized that the changes in intracellular hypotaurine levels would be reflected in the effects on tumor metabolites, and this was supported by our results of hypotaurine’s contribution to cellular invasion, proliferation, and apoptosis. We also conjecture that the inhibitory effects of taurine are mediated by the ERK/RSK cascade *via* the EMT and by taurine’s competitive inhibition of hypotaurine.

Consistent with our *in vitro* results of taurine-attenuated, hypotaurine-induced tumor growth, taurine significantly decreased the growth of HT-29 and LoVo xenograft tumors, laying the foundation for further rational treatment of this kind in CRCs. In addition, the use of taurine can increase apoptosis and decrease proliferation *in vivo*. We herein also demonstrated that taurine inhibited cellular migration, invasion, and metastasis *in vitro* and *in vivo*. Orthotopic transplantation of CT26 cells into taurine-treated mice did not produce liver or lung metastasis, and this was supported by the number of metastatic nodules and the expression of bioluminescent signals. Therefore, taurine treatment can prevent the EMT and inhibit CRC cell dissemination. Metabonomics has been widely used in uncovering biomarkers due to its close relationship with phenotype and sensitivity to many factors. As a “downstream” type of genomics, transcriptomics, and proteomics, metabonomics has been used to detect a variety of subtle modifications that reflect changes in biologic state, even if there are no measurable changes in corresponding genes and proteins. Therefore, candidates for potential biomarkers are more likely to be made available through metabonomics (Monteiro et al., 2013). By comparison, endogenous metabolites number fewer than genes, transcripts, and proteins, have the same basic chemical structures, and are extremely conserved, making it simpler to analyze metabolic results. Furthermore, metabonomics can be used for non-invasive assessments of biologic samples, and can be more easily translated into clinical practice. These metabolic abnormalities driven by cancer may represent the inherent characteristics of the tumor, and may even predict the pathogenesis of cancer, providing changes in serum metabolites related to systematic phenotypic biology (Tian et al., 2018). Therefore, changes in serum metabolites may reveal unique pathologic conditions, and provide early detection and prognosis. Our results showed that serum taurine could be used to differentiate CRC patients from healthy controls, and that the low taurine levels observed in CRC were correlated with metastases to lung and liver. Our multivariate analyses also indicated that a low level of taurine might be an independent risk factor in the progression of CRC.

In conclusion, we herein demonstrated that hypotaurine is involved in CRC progression, and that taurine attenuates this tendency. The inhibitory effects of taurine on CRC progression are likely mediated by the ERK/RSK cascade *via* the EMT, and by taurine’s competitive inhibition of hypotaurine. Our results indicated that taurine may constitute a promising agent in the treatment of CRC. Our results also provided evidence of the interaction among metabolism, signal transduction, and tumor pathophysiology. Taurine attenuated hypotaurine-induced tumor invasion and proliferation, and this aspect provides a promising target for early diagnosis and therapy. It is, however, necessary to further study the role of taurine in order to provide a potential translational tool for CRC treatments.

## Data Availability Statement

The raw data supporting the conclusions of this article will be made available by the authors, without undue reservation.

## Ethics Statement

The studies involving human participants were reviewed and approved by Shanghai Jiao Tong University School of Medicine, Renji Hospital Ethics Committee. The patients/participants provided their written informed consent to participate in this study. The animal study was reviewed and approved by Shanghai Jiao Tong University School of Medicine, Renji Hospital Ethics Committee.

## Author Contributions

XH, JH, and XZ designed the project and performed most of the experiments. QW and RZ contributed to acquisition of clinical data. QL and ML wrote the main manuscript text and supervised the entire project. All authors contributed to manuscript revision, read, and approved the submitted version.

## Conflict of Interest

The authors declare that the research was conducted in the absence of any commercial or financial relationships that could be construed as a potential conflict of interest.

## Footnotes

1 http://www.genome.jp/kegg/

2 http://www.hmdb.ca/

3 http://www.massbank.jp/
