# Supplementary material for: Taurine Attenuates the Hypotaurine-Induced Progression of CRC via ERK/RSK Signaling
Source: Front Cell Dev Biol. 2021 Apr 15;9:631163. doi: 10.3389/fcell.2021.631163 (PMC8083965; doi:10.3389/fcell.2021.631163)
Supplement: Supplementary file 1 [file Data_Sheet_1.PDF]

Supplementary Material

1 SUPPLEMENTARY FIGURES

A

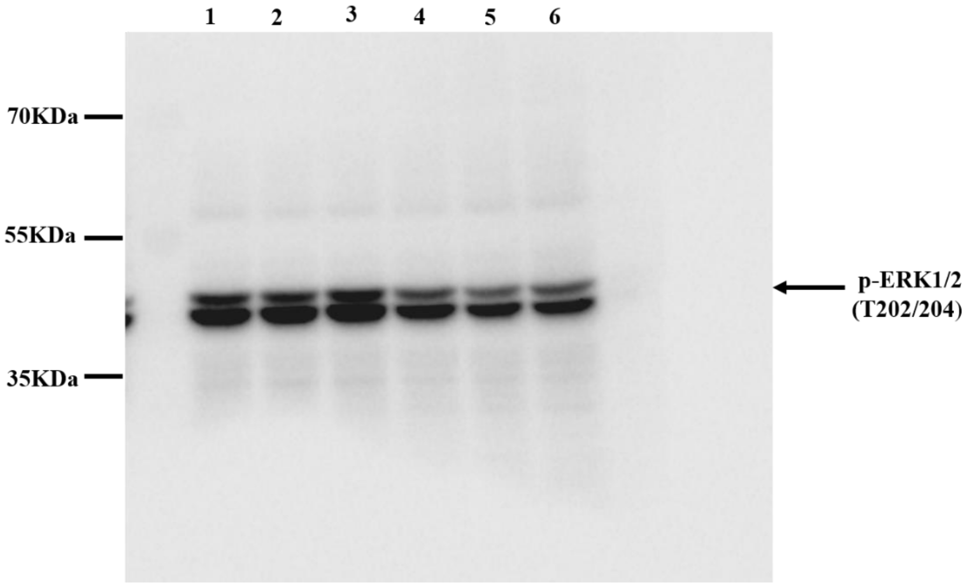

B

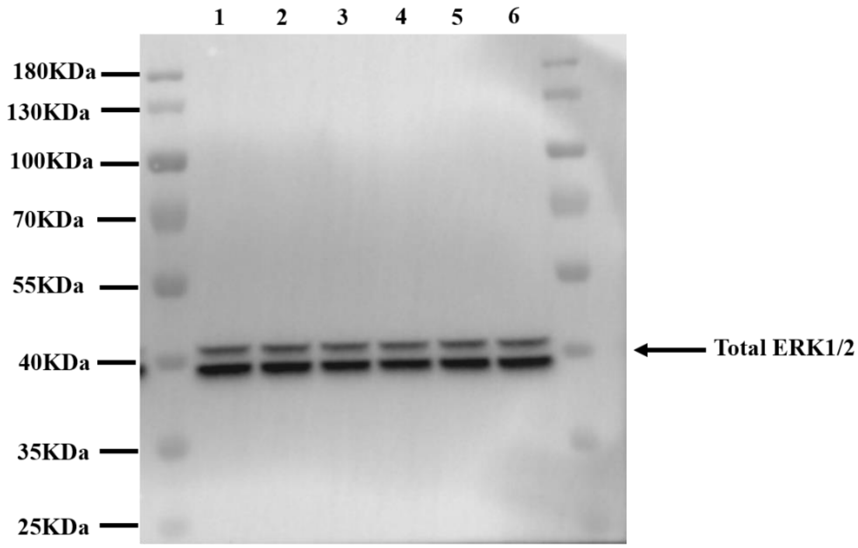

C

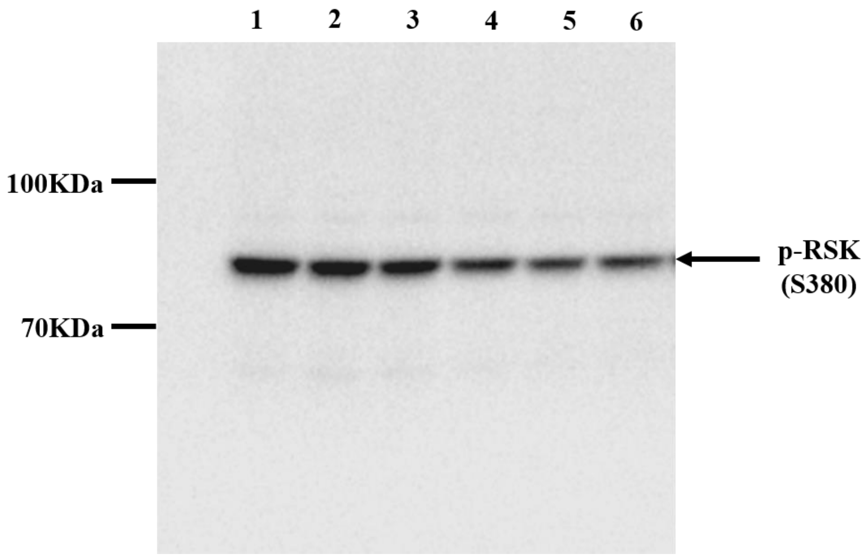
1
2
3
4
5
6
100KDa
70KDa
p-RSK
(S380)

D

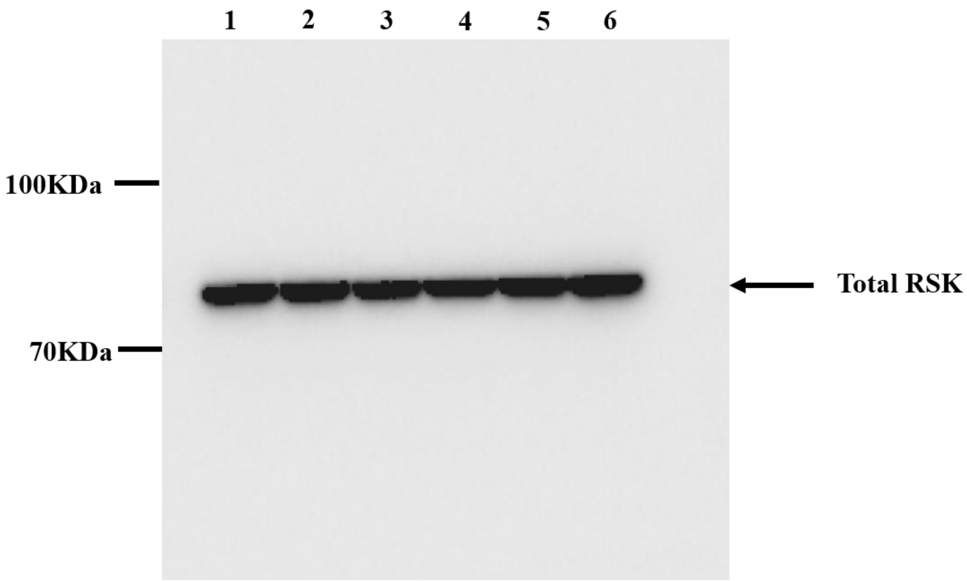
1
2
3
4
5
6
100KDa
70KDa
Total RSK

E

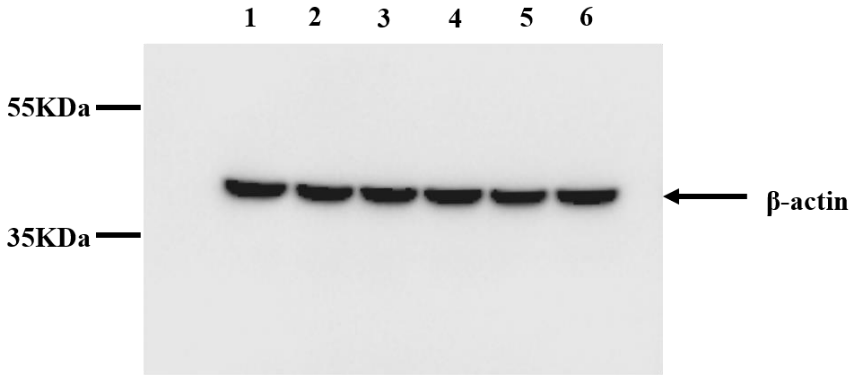
1
2
3
4
5
6
55KDa
35KDa
β-actin

SUPPLEMENTARY FIGURE 1. The complete, unedited WB gels for Figure 5A (HT-29). (1) Taurine 0 mM **(2)** Taurine 1.25 mM **(3)** Taurine 2.5 mM **(4)** Taurine 5 mM **(5)** Taurine 10 mM **(6)** Taurine 20 mM.

A

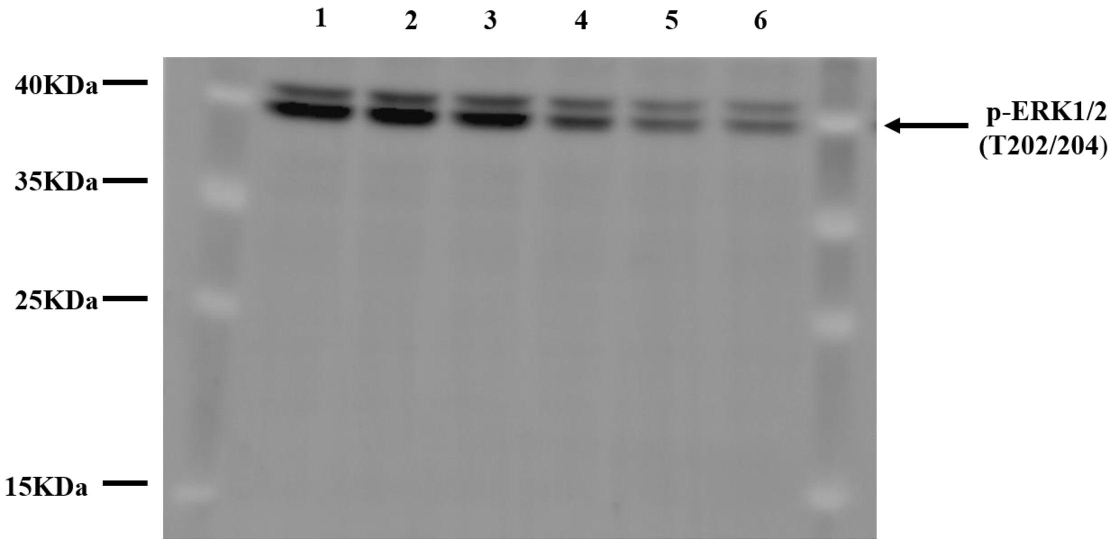

B

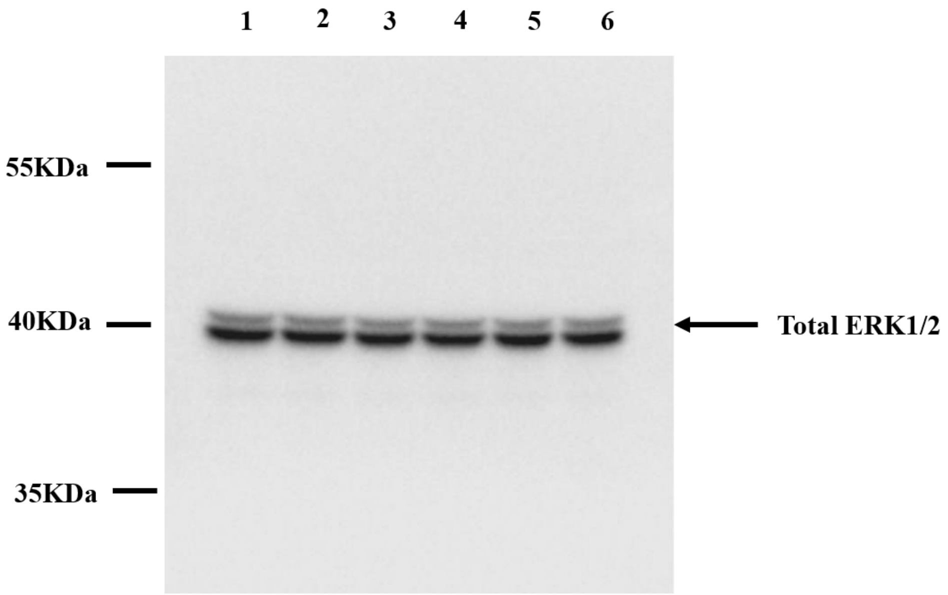

C

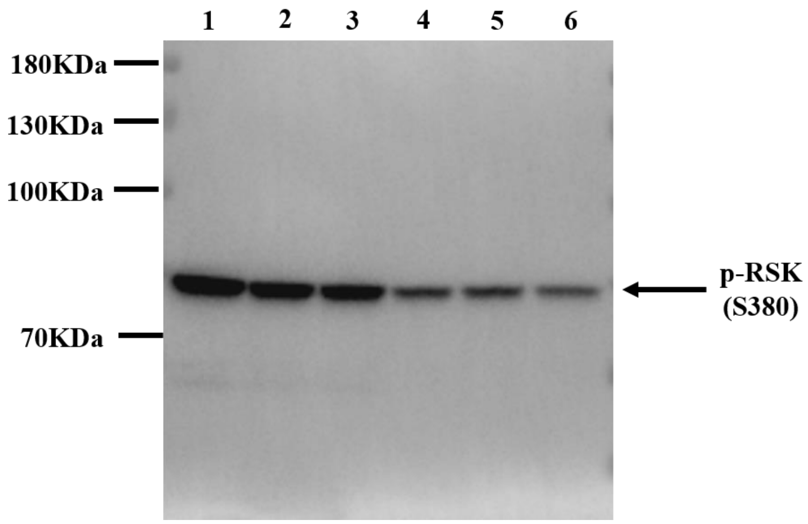

D

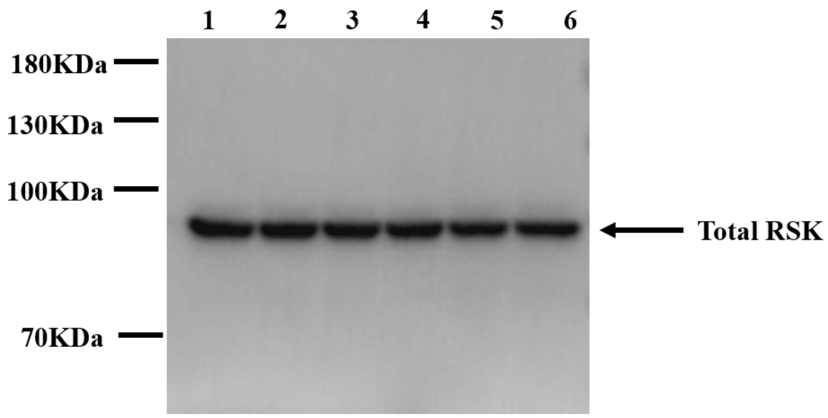

E

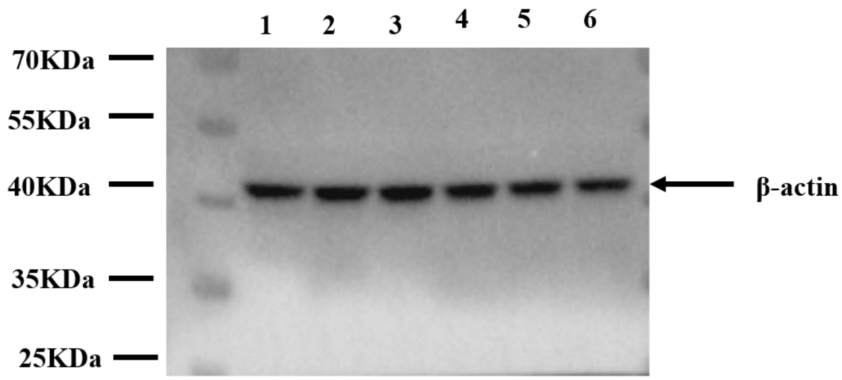

SUPPLEMENTARY FIGURE 2. The complete, unedited WB gels for Figure 5A (LoVo). (1) Taurine 0 mM **(2)** Taurine 1.25 mM **(3)** Taurine 2.5 mM **(4)** Taurine 5 mM **(5)** Taurine 10 mM **(6)** Taurine 20 mM.

A

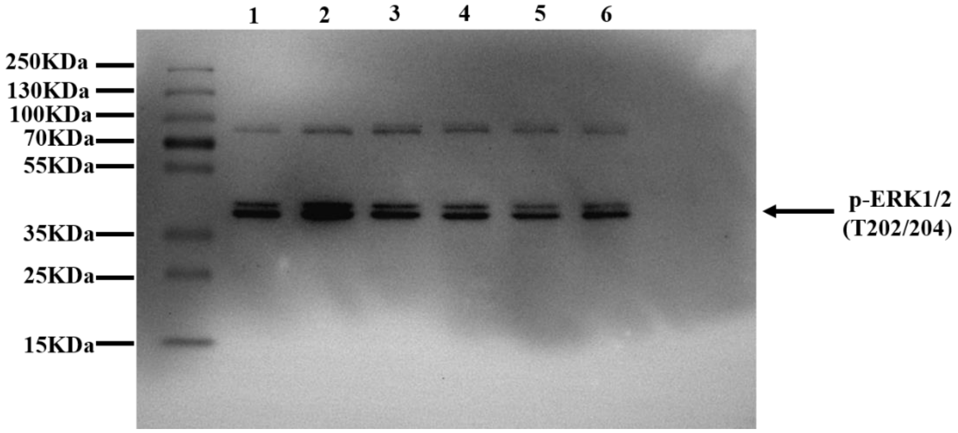
1
2
3
4
5
6
250KDa
130KDa
100KDa
70KDa
55KDa
35KDa
25KDa
15KDa
p-ERK1/2
(T202/204)

B

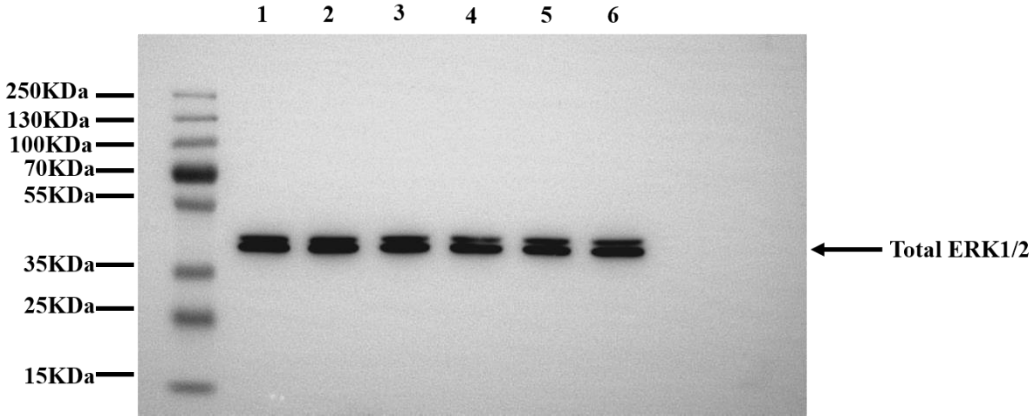
1
2
3
4
5
6
250KDa
130KDa
100KDa
70KDa
55KDa
35KDa
25KDa
15KDa
Total ERK1/2

C

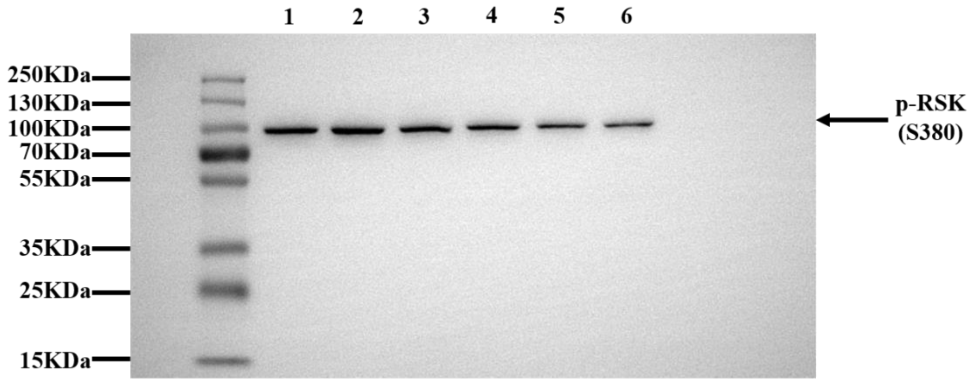
1
2
3
4
5
6
250KDa
130KDa
100KDa
70KDa
55KDa
35KDa
25KDa
15KDa
p-RSK
(S380)

D

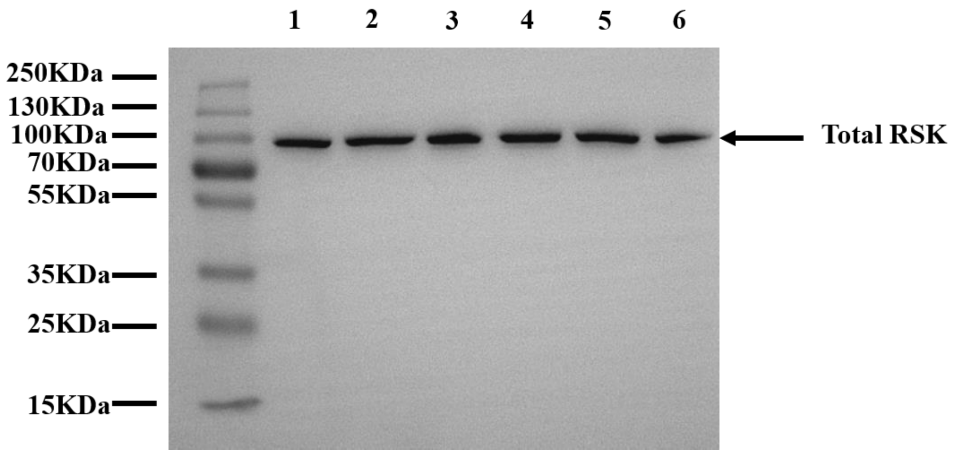

E

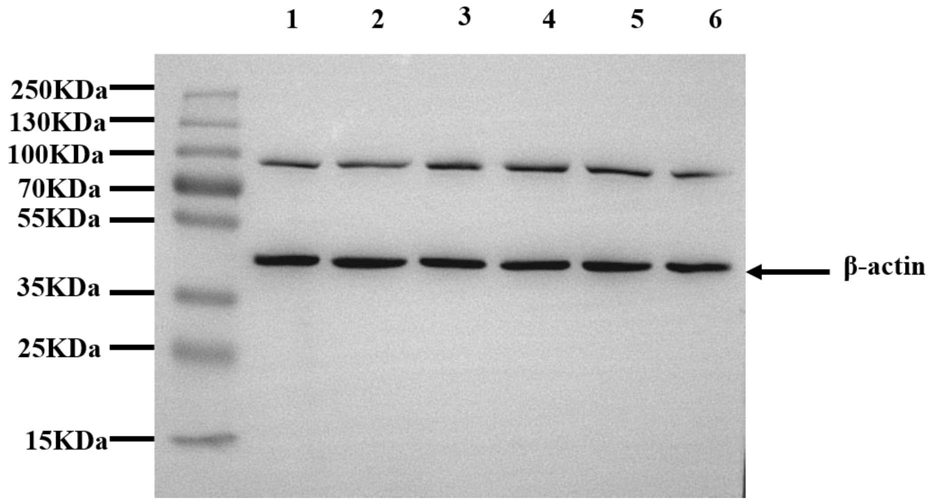

SUPPLEMENTARY FIGURE 3. The complete, unedited WB gels for Figure 5B (HT-29). (1) Taurine 0 mM+Hypotaurine 0 mM **(2)** Taurine 0 mM+Hypotaurine 10 mM **(3)** Taurine 2.5 mM+Hypotaurine 10 mM **(4)** Taurine 5 mM+Hypotaurine 10 mM **(5)** Taurine 10 mM+Hypotaurine 10 mM **(6)** Taurine 20 mM+Hypotaurine 10 mM.

A

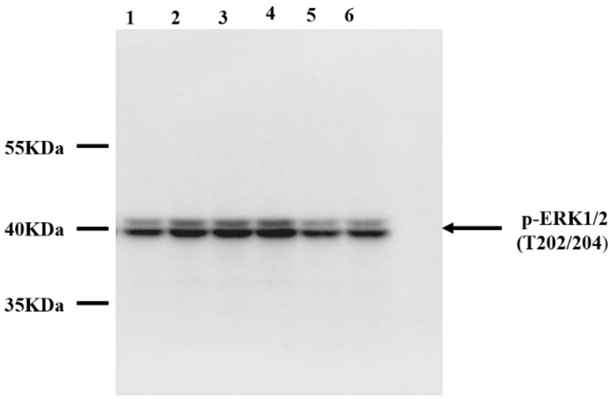

B

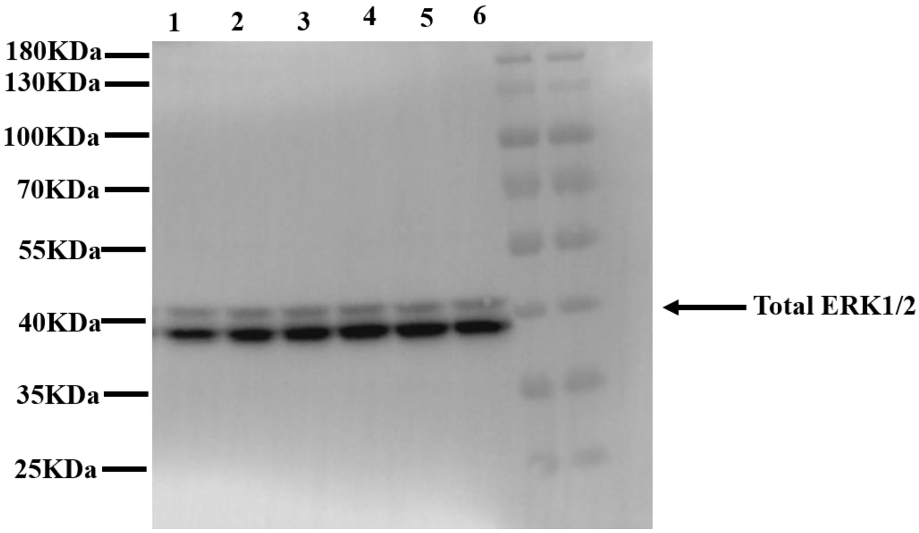
1
2
3
4
5
6
180KDa
130KDa
100KDa
70KDa
55KDa
40KDa
35KDa
25KDa
Total ERK1/2

C

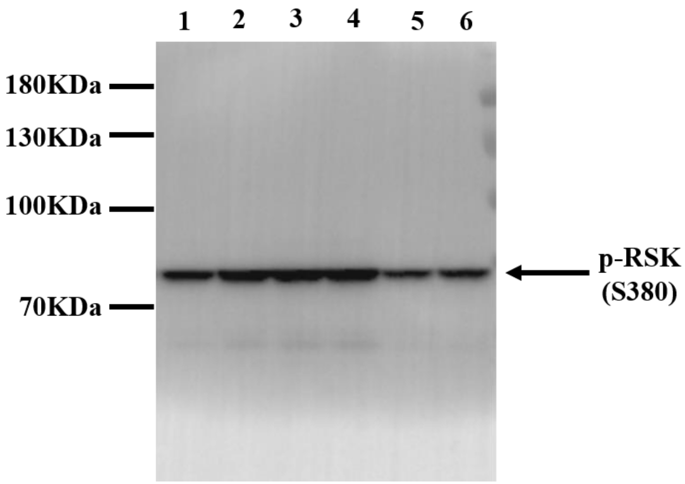
1
2
3
4
5
6
180KDa
130KDa
100KDa
70KDa
p-RSK
(S380)

D

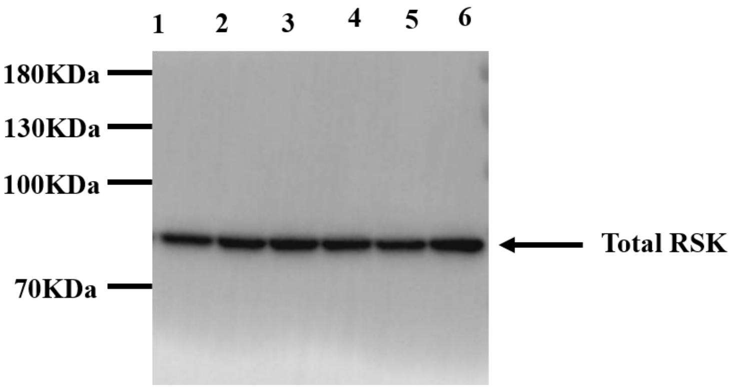
1
2
3
4
5
6
180KDa
130KDa
100KDa
70KDa
Total RSK

E

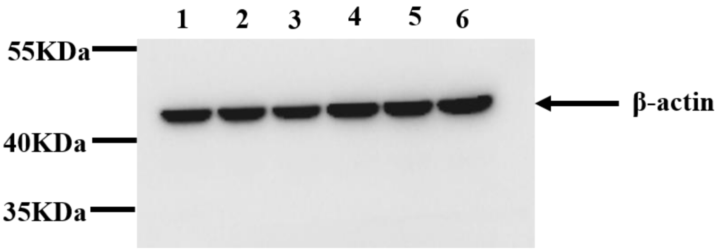

SUPPLEMENTARY FIGURE 4. The complete, unedited WB gels for Figure 5B (LoVo). (1) Taurine 0 mM+Hypotaurine 0 mM **(2)** Taurine 0 mM+Hypotaurine 10 mM **(3)** Taurine 2.5 mM+Hypotaurine 10 mM **(4)** Taurine 5 mM+Hypotaurine 10 mM **(5)** Taurine 10 mM+Hypotaurine 10 mM **(6)** Taurine 20 mM+Hypotaurine 10 mM.

A

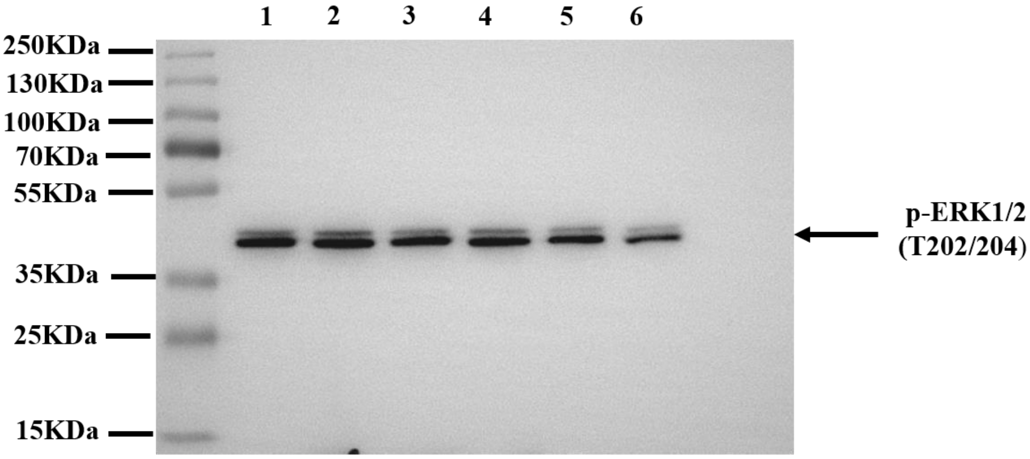

B

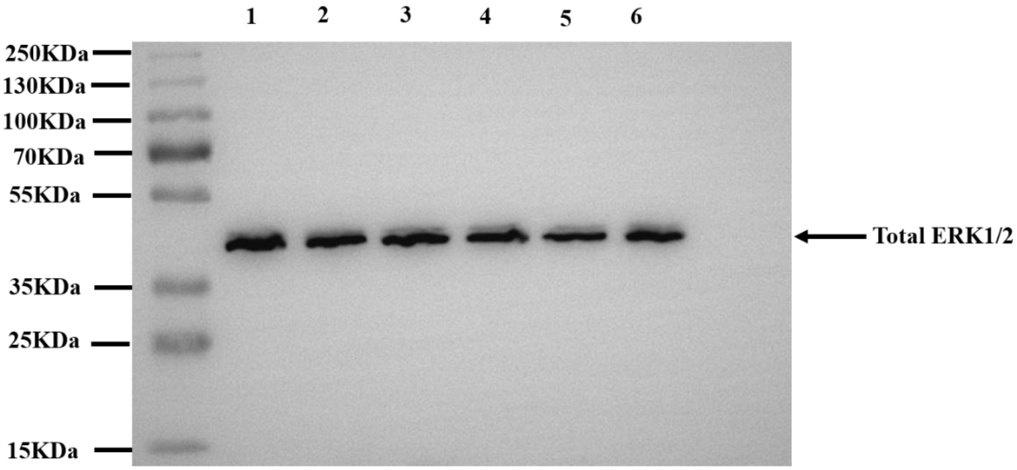

C

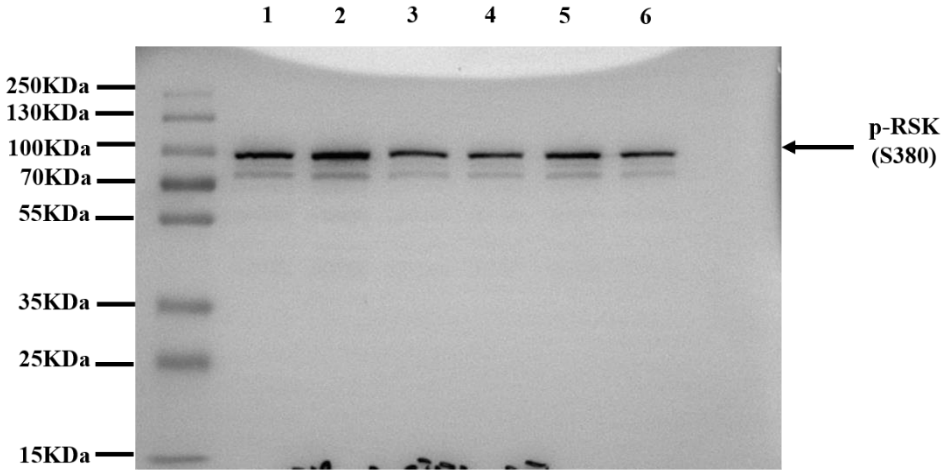
1
2
3
4
5
6
250KDa
130KDa
100KDa
70KDa
55KDa
35KDa
25KDa
15KDa
p-RSK
(S380)

D

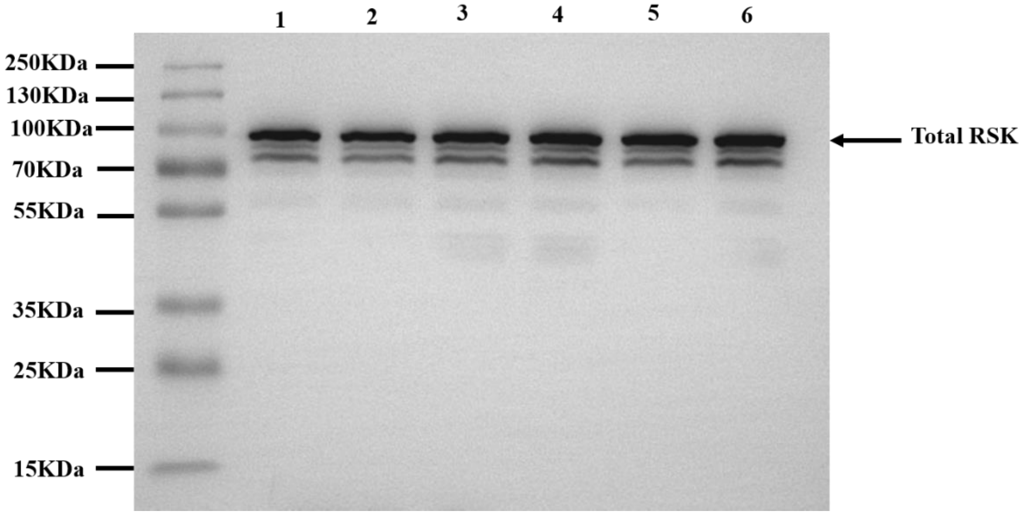
1
2
3
4
5
6
250KDa
130KDa
100KDa
70KDa
55KDa
35KDa
25KDa
15KDa
Total RSK

E

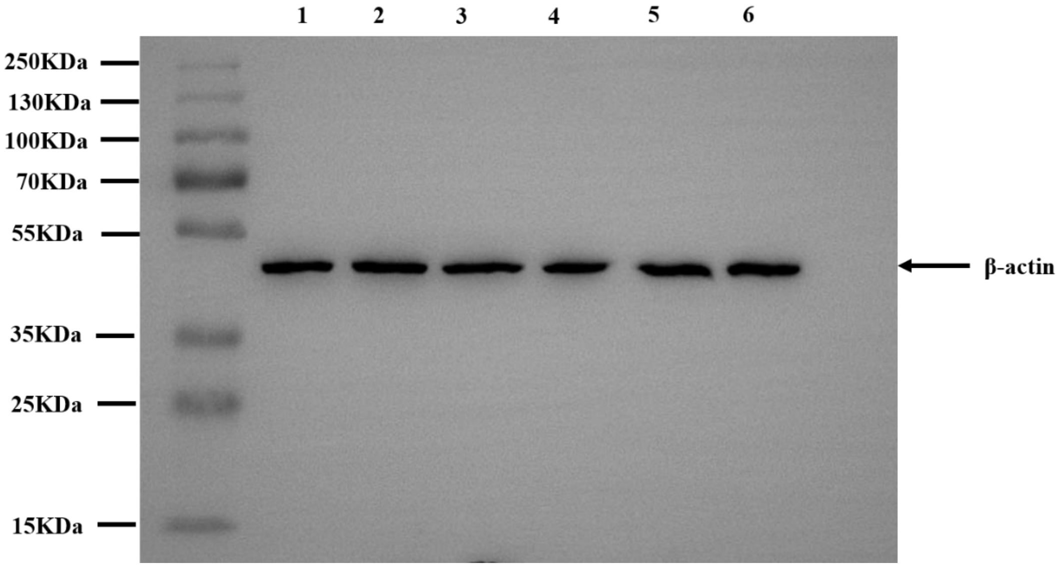
1
2
3
4
5
6
250KDa
130KDa
100KDa
70KDa
55KDa
35KDa
25KDa
15KDa
β-actin

SUPPLEMENTARY FIGURE 5. The complete, unedited WB gels for Figure 6A (HT-29). (1) SCH772984 0 µM+Hypotaurine 0 mM **(2)** SCH772984 0 µM+Hypotaurine 10 mM **(3)** SCH772984 0.5 µM+Hypotaurine 10 mM **(4)** SCH772984 1 µM+Hypotaurine 10 mM **(5)** SCH772984 2 µM +Hypotaurine 10 mM **(6)** SCH772984 4 µM+Hypotaurine 10 mM.

A

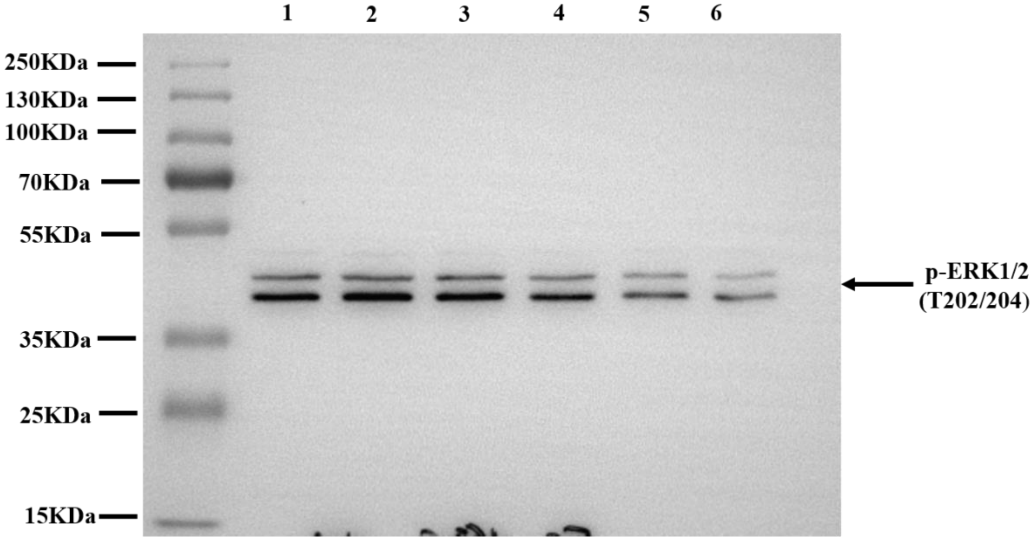

B

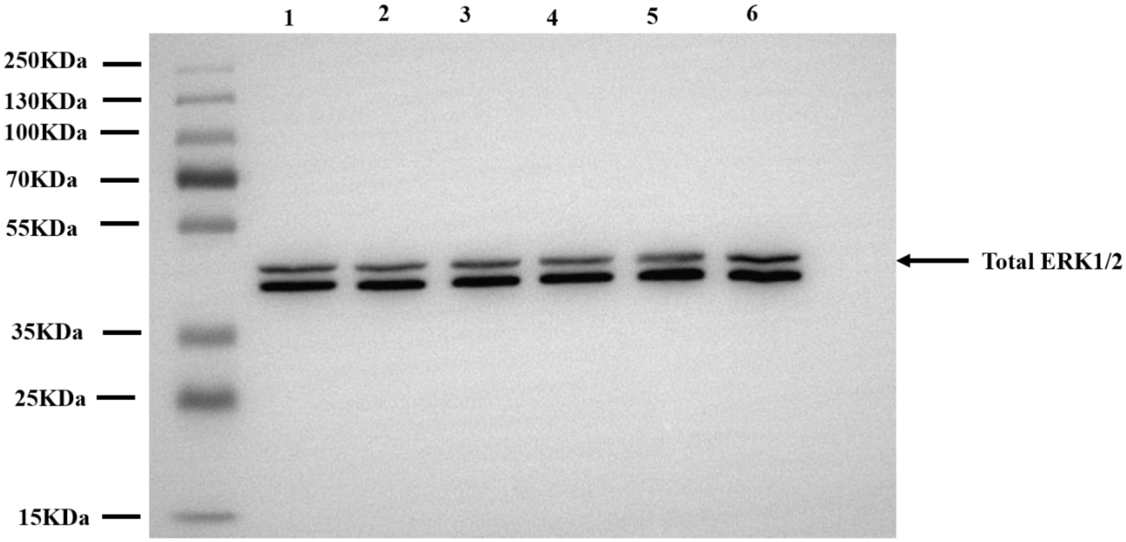

C

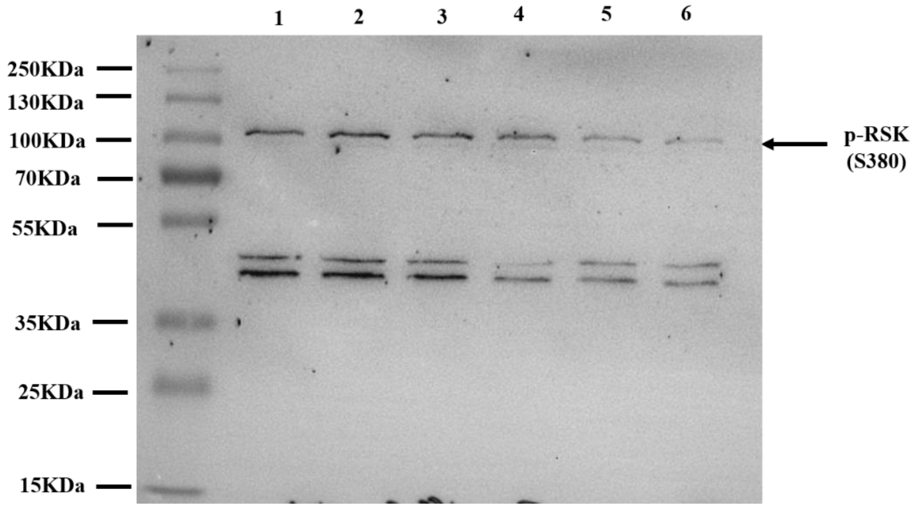
1
2
3
4
5
6
250KDa
130KDa
100KDa
70KDa
55KDa
35KDa
25KDa
15KDa
p-RSK
(S380)

D

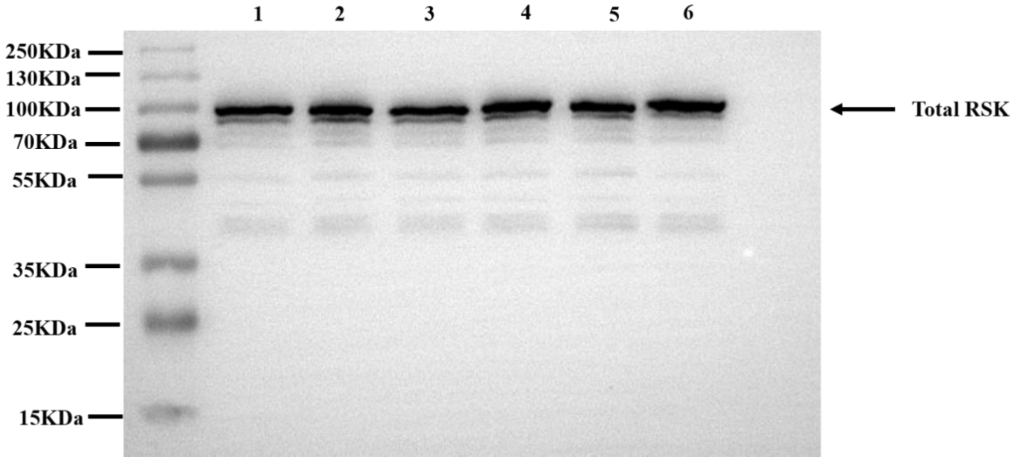
1
2
3
4
5
6
250KDa
130KDa
100KDa
70KDa
55KDa
35KDa
25KDa
15KDa
Total RSK

E

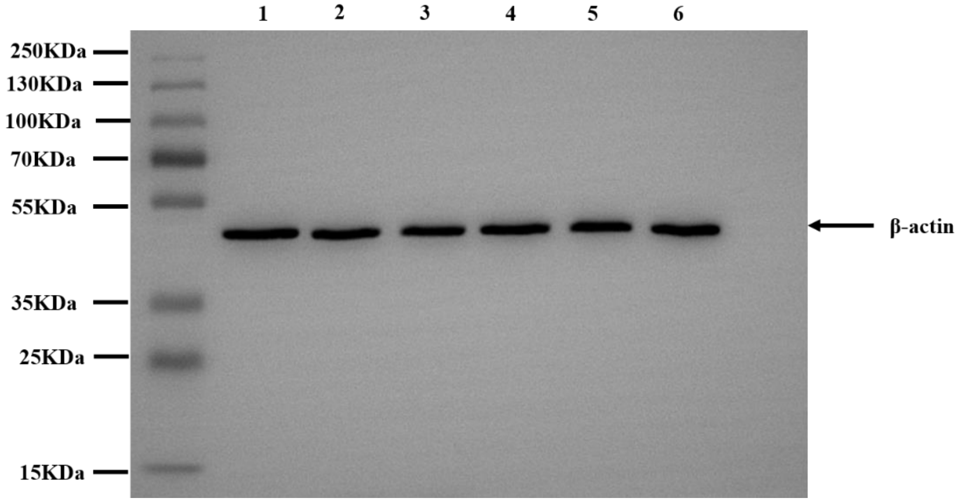
1
2
3
4
5
6
250KDa
130KDa
100KDa
70KDa
55KDa
35KDa
25KDa
15KDa
β-actin

SUPPLEMENTARY FIGURE 6. The complete, unedited WB gels for Figure 6A (LoVo). (1) SCH772984 0 μM+Hypotaurine 0 mM **(2)** SCH772984 0 μM+Hypotaurine 10 mM **(3)** SCH772984 0.5 μM+Hypotaurine 10 mM **(4)** SCH772984 1 μM+Hypotaurine 10 mM **(5)** SCH772984 2 μM +Hypotaurine 10 mM **(6)** SCH772984 4 μM+Hypotaurine 10 mM.

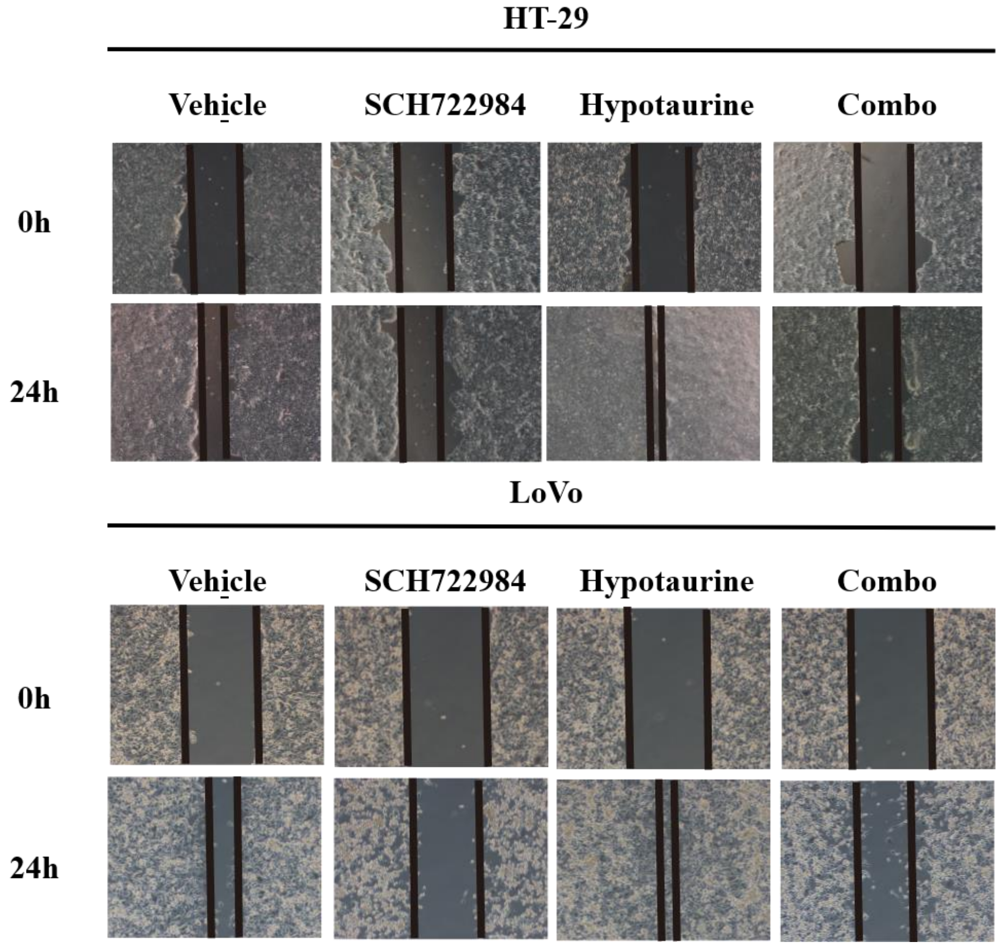

SUPPLEMENTARY FIGURE 7. Representative photomicrographs of cellular migration induced by SCH772984 and/or hypotaurine.

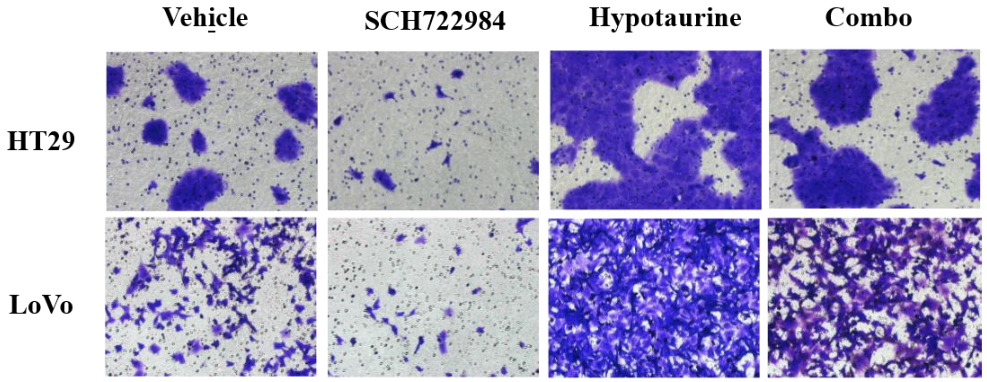

SUPPLEMENTARY FIGURE 8. Representative photomicrographs of cellular invasion induced

by SCH772984 and/or hypotaurine.

A

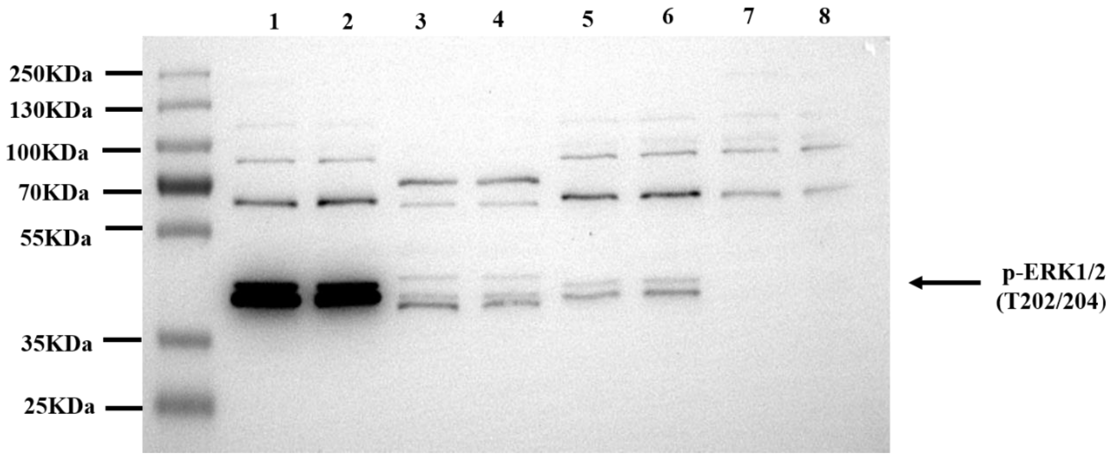

B

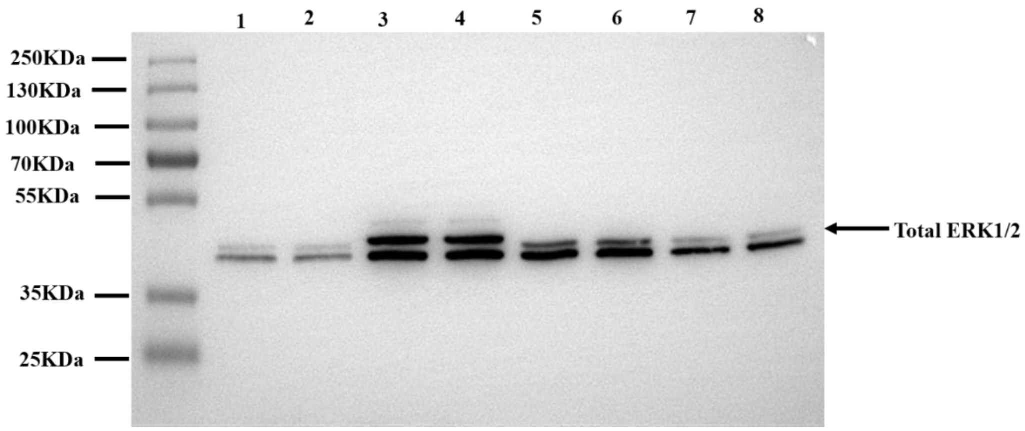

C

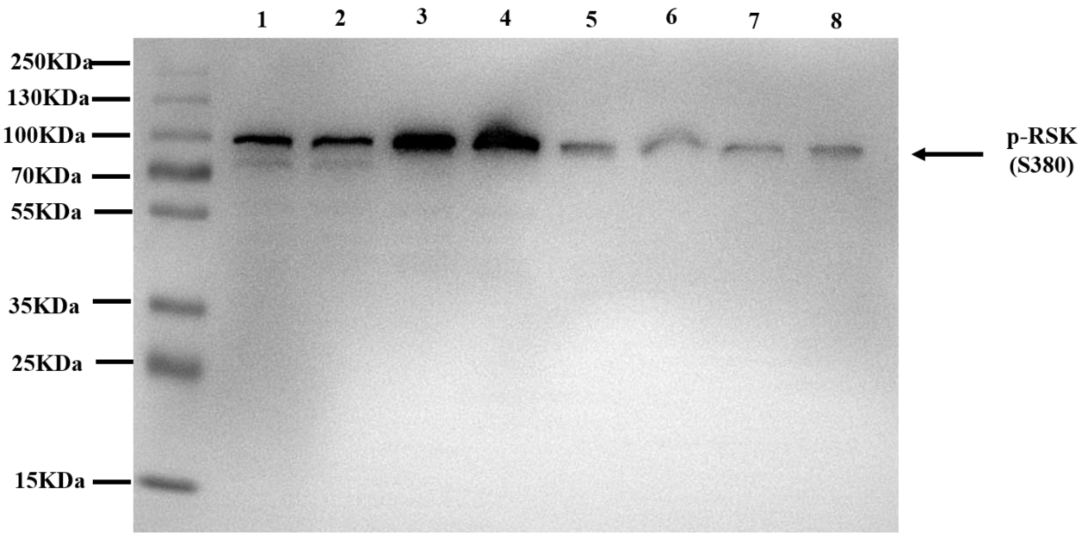

D

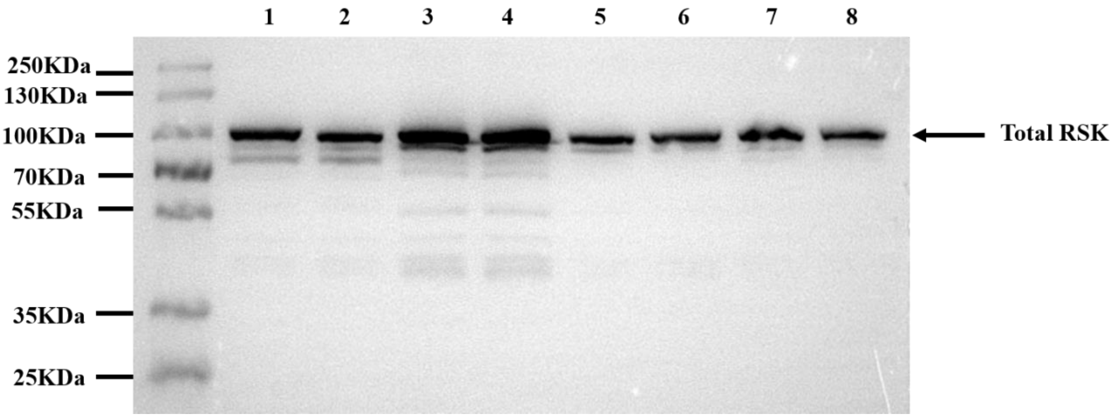

E

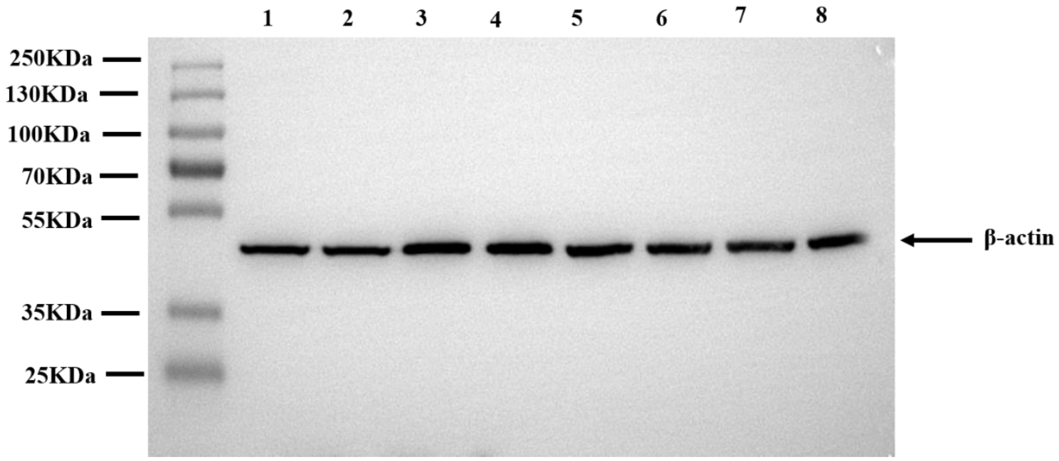

SUPPLEMENTARY FIGURE 9. The effects of vehicle on the phosphorylation (denoted p-) status of ERK and RSK as detected by western immunoblotting analysis. (1) HT-29 cells treated with vehicle (0 mM) **(2)** HT-29 cells treated with vehicle (10 mM) **(3)** LoVo cells treated with vehicle (0 mM) **(4)** LoVo cells treated with vehicle (10 mM) **(5)** SW480 cells treated with vehicle (0 mM) **(6)** SW480 cells treated with vehicle (10 mM) **(7)** SW620 cells treated with vehicle (0 mM) **(8)** SW620 cells treated with vehicle (10 mM).

2 SUPPLEMENTARY TABLES

SUPPLEMENTARY TABLE 1. Clinicopathologic characteristics of all colon cancer patients analyzed (N=42).

Characteristics	N=42
**Sex**	
Male	25
Female	17

**Age, years**	
Median	55
Range	35-80
**Clinical Stage**	
Stage I	8
Stage II	12
Stage III	15
Stage IV	7
**T Stage**	
T1	6
T2	18
T3	14
T4	4
**N Stage**	
N0	21
N1	15
N2	6
**M Stage**	
M0	35
M1	7
